# Kinetic Investigations of the Role of Factor Inhibiting Hypoxia-inducible Factor (FIH) as an Oxygen Sensor*[Fn FN2]

**DOI:** 10.1074/jbc.M115.653014

**Published:** 2015-06-25

**Authors:** Hanna Tarhonskaya, Adam P. Hardy, Emily A. Howe, Nikita D. Loik, Holger B. Kramer, James S. O. McCullagh, Christopher J. Schofield, Emily Flashman

**Affiliations:** From the ‡ Chemistry Research Laboratory, University of Oxford, 12 Mansfield Road, Oxford OX1 3TA, United Kingdom and; the § OXION Proteomics Facility, Department of Physiology, Anatomy, and Genetics, University of Oxford, South Parks Road, Oxford OX1 3QX, United Kingdom

**Keywords:** dioxygenase, hypoxia, hypoxia-inducible factor (HIF), kinetics, pre-steady-state kinetics, HIF hydroxylase, ankyrin repeat domain, factor inhibiting HIF, oxygen sensor, prolyl hydroxylase

## Abstract

The hypoxia-inducible factor (HIF) hydroxylases regulate hypoxia sensing in animals. In humans, they comprise three prolyl hydroxylases (PHD1–3 or EGLN1–3) and factor inhibiting HIF (FIH). FIH is an asparaginyl hydroxylase catalyzing post-translational modification of HIF-α, resulting in reduction of HIF-mediated transcription. Like the PHDs, FIH is proposed to have a hypoxia-sensing role in cells, enabling responses to changes in cellular O_2_ availability. PHD2, the most important human PHD isoform, is proposed to be biochemically/kinetically suited as a hypoxia sensor due to its relatively high sensitivity to changes in O_2_ concentration and slow reaction with O_2_. To ascertain whether these parameters are conserved among the HIF hydroxylases, we compared the reactions of FIH and PHD2 with O_2_. Consistent with previous reports, we found lower *K*_*m*_^app^(O_2_) values for FIH than for PHD2 with all HIF-derived substrates. Under pre-steady-state conditions, the O_2_-initiated FIH reaction is significantly faster than that of PHD2. We then investigated the kinetics with respect to O_2_ of the FIH reaction with ankyrin repeat domain (ARD) substrates. FIH has lower *K*_*m*_^app^(O_2_) values for the tested ARDs than HIF-α substrates, and pre-steady-state O_2_-initiated reactions were faster with ARDs than with HIF-α substrates. The results correlate with cellular studies showing that FIH is active at lower O_2_ concentrations than the PHDs and suggest that competition between HIF-α and ARDs for FIH is likely to be biologically relevant, particularly in hypoxic conditions. The overall results are consistent with the proposal that the kinetic properties of individual oxygenases reflect their biological capacity to act as hypoxia sensors.

## Teaser

**Background:** The hypoxia-inducible factor (HIF) hydroxylases (FIH and PHDs) regulate hypoxia sensing in animals.

**Results:** FIH·HIF-α reacts faster with O_2_ than PHD2·HIF-α but slower than FIH·ankyrin complexes.

**Conclusion:** The kinetics of catalysis by isolated FIH and PHD2 reflect their cellular activities.

**Significance:** We provide a kinetic rationale for cellular observations that FIH activity is more hypoxia-tolerant than that of the PHDs.

## Introduction

The chronic hypoxic response in animals is mediated by the heterodimeric (α/β) hypoxia-inducible transcriptional factor (HIF),^3^ accumulation of which triggers up-regulation of an array of genes, including those encoding for erythropoietin, vascular endothelial growth factor (VEGF), vasomotor regulators, and glycolytic enzymes (reviewed in Refs. 1–4). The levels and activity of HIF isoforms in cells are regulated by post-translational hydroxylation of their HIF-α domains, as catalyzed by prolyl hydroxylase domain enzymes (PHD1–3, also known as EGLN1–3) and factor inhibiting HIF (FIH), collectively termed the HIF hydroxylases (5). The PHDs catalyze hydroxylation of one or both of two proline residues in the O_2_-dependent degradation region of HIF-α (C-terminal oxygen-dependent degradation domain (CODD) and N-terminal oxygen-dependent degradation domain (NODD)), resulting in its targeting to the proteasome (2, 6). FIH catalyzes hydroxylation of an asparaginyl residue in the HIF-α C-terminal transactivation domain (CAD), which leads to reduced interaction of HIF with the transcriptional co-activator proteins CBP/p300 (CREB-binding protein/p300) (7–9). Together with the PHDs, FIH is proposed to act as an O_2_ sensor in cells, providing a direct link between cellular O_2_ concentrations and the hypoxic response (5, 10, 11).

The PHDs and FIH catalyze hydroxylation of both HIF-1α and HIF-2α isoforms. The HIF-1/2α isoforms have similar domain architectures and characteristics; however, differences in their properties have been identified, and they are not biologically redundant (12, 13). Whereas HIF-1α is mostly responsible for activation of glycolytic pathway genes (*e.g. HK1* (hexokinase 1) and *HK2* (hexokinase 2)), HIF-2α is involved in regulation of genes responsible for cell cycle progression (*e.g.* cyclin D1), tumor growth, erythropoiesis (*EPO* (erythropoietin)), and maintaining stem cell pluripotency among other functions (14, 15). In addition, there is evidence that HIF-1/2α can act as either pro-oncogenic or tumor suppressor factors, depending on the context in which they are present (12, 16, 17).

Although FIH was first identified as a HIF hydroxylase, it has subsequently been shown to hydroxylate semiconserved Asn residues in many proteins containing ankyrin repeat domains (ARDs) (18). ARDs comprise a conserved sequence of 33 residues and are extensively involved in protein-protein interactions (reviewed in Ref. 19). The range of ARD-containing substrates for FIH is apparently very diverse (19), including tankyrase, NF-κB (nuclear factor κ-light chain enhancer of activated B cells, a protein complex that controls transcription of DNA), Notch-1 (neurogenic locus notch homolog protein, a transmembrane protein involved in signaling), MYPT-1 (the myosin phosphatase targeting protein, a regulatory subunit of protein phosphatase 1), and vanilloid 3 channel (hydroxylation of which abolishes activity (20)). In contrast to the “switchlike” signaling effect of CAD hydroxylation on HIF activity, no such well defined role for FIH-catalyzed ARD hydroxylation has yet been defined. In some but probably not all cases, Asn hydroxylation has been observed to stabilize the ARD protein fold (21–24). Competition between ARDs and HIF-α isoforms for FIH is proposed to tune the hypoxic response and has the potential to enable memory of hypoxic events (25). Recombinant FIH has been shown to be capable of catalyzing hydroxylation of residues besides Asn in the context of ARDs (*e.g.* His, Leu, or Ser residues) (26), with some of these reactions having been shown to occur in human cells (27), demonstrating that FIH is a highly promiscuous enzyme.

**FIGURE 1.**
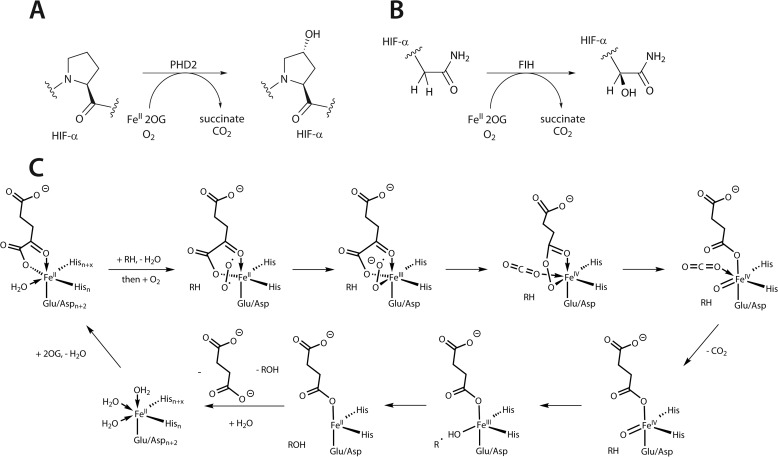
**The PHD- and FIH-catalyzed reactions and outline of the proposed consensus mechanism for 2OG-dependent oxygenases.** *A*, prolyl hydroxylation performed by PHD2; *B*, asparaginyl hydroxylation as performed by FIH; *C*, O_2_ binding subsequent to formation of the enzyme·substrate complex leads to 2OG decarboxylation with the formation of an Fe(IV)-oxo intermediate, which then performs oxidative modification of the “prime” substrate (*e.g.* HIF-α).

The HIF hydroxylases are Fe(II)/2-oxoglutarate (2OG)-dependent oxygenases, a family of enzymes with wide-ranging biological roles (28). The 2OG oxygenases have a conserved double-stranded β-helix fold that supports an Fe(II)-coordinating H*X*(D/E)*X_n_*H catalytic triad and employ a conserved consensus oxidation mechanism (29, 30): formation of an enzyme·Fe(II)·2OG·substrate ternary complex allows O_2_ activation followed by 2-electron oxidation of 2OG coupled to oxidation of the substrate (Fig. 1). A substantial body of structural, kinetic, and spectroscopic evidence supports this consensus mechanism (29, 31–34). Transient kinetics combined with spectroscopic techniques have provided evidence for intermediates (including the reactive Fe(IV)-oxo species (32)) and have been used to measure the kinetics of events following O_2_ binding. For some 2OG oxygenases, O_2_ binding is followed by rapid formation of the reactive intermediates (∼<1 s) (*e.g.* for TauD (taurine dioxygenase) (32, 35), vCPH (a viral collagen prolyl hydroxylase) (36), and DAOCS (deacetoxycephalosporin C synthase) (37). However, for PHD2, the most important of the human PHDs, we found that the rate of reaction subsequent to O_2_ exposure was considerably slower than for TauD (∼100-fold) (38). Combined with the graded response of PHD2 to increasing O_2_ concentrations in steady-state kinetic assays and also in cells (11, 39), we have proposed that the apparently slow reaction of PHD2 with O_2_ reflects its role as an O_2_ sensor (38).

Although FIH is proposed to be an O_2_-sensing HIF hydroxylase, cellular and kinetic data suggest that FIH is less efficient at detecting deviations from normoxia than the PHDs. In cells, FIH is still substantially active under hypoxic conditions, whereas PHD activity drops significantly (39). The differences are apparently reflected in the lower reported *K*_*m*_^app^(O_2_) values for FIH (∼90–200 μm for FIH compared with ∼250 μm to 1.7 mm for PHD2 (10, 40–42)). Interestingly, the *K*_*m*_^app^(O_2_) for FIH has also been reported to be substrate-dependent (43), with a lower *K*_*m*_^app^ for O_2_ for Notch-1 (ARD) hydroxylation than for HIF-1α hydroxylation, suggesting that FIH may be more active toward at least some ARDs than HIF-α substrates in hypoxia.

Because targeting the HIF hydroxylases for the treatment of hypoxia-related diseases is of medicinal interest (PHD inhibitors are presently undergoing large scale clinical trials), a detailed understanding of the kinetics of these enzymes with respect to O_2_ is important to compare and rationalize the sensitivity of the HIF hydroxylases to hypoxia. Following our observation of the slow reaction of PHD2 with O_2_, we therefore conducted a detailed investigation of the kinetics of the reaction of FIH with O_2_. First, we investigated whether the slow reaction with O_2_ (as observed for a PHD2·HIF-1α complex (38, 42)) was conserved among O_2_-sensing HIF hydroxylases. Second, we investigated the effect of different substrates on the kinetics of the reactions of PHD2 and FIH with O_2_, in terms of HIF-1/2α isoforms, HIF hydroxylation sites for PHD2, and FIH with HIF *versus* ARD substrates. We describe spectroscopic, kinetic, and mechanistic evidence revealing that the reaction of PHD2 with O_2_ is notably slow in the presence of all tested HIF substrates. We also report that the reaction of FIH with O_2_ is significantly faster than for PHD2 in the presence of all HIF-1/2α substrates and to an even greater degree in the presence of all tested ARD substrates. The results are consistent with studies indicating that FIH is more active than the PHDs under hypoxic conditions (39) as well as the reported ability of FIH to preferentially hydroxylate ARD rather than HIF substrates (19, 44). The work also demonstrates that, at least for PHD2 and FIH, the kinetic properties of these enzymes with respect to O_2_ reflect their cellular hypoxia-sensing ability.

## Experimental Procedures

Full-length recombinant FIH, two- and three-consensus ankyrin repeats (2CA and 3CA) were produced as described (21) in *Escherichia coli* BL21(DE3) cells and purified as His_6_-tagged proteins with subsequent thrombin cleavage of the tag. The catalytic domain of PHD2 (residues 181–426) was purified as described (45). 1CA, tankyrase-1_20-mer_, HIF-2α CAD_35-mer_, HIF-1α CAD_35-mer_, tankyrase-1_35-mer_, HIF-1α CAD_19-mer_, HIF-2α CAD_19-mer_, HIF-1α or HIF-2α CODD_19-mer_, and HIF-1α or HIF-2α NODD_19-mer_ peptides were purchased from GL Biochem (Shanghai) Ltd., China. The N and C termini of the peptides were amino groups and carboxylates (Table 1).

### 

#### 

##### Enzyme Activity Assays and Studies of O_2_ Dependence

Enzymatic assays were conducted as described (46, 47). Unless otherwise stated, the assay mixture typically contained 5 μm FIH, 500 μm substrate, 500 μm 2OG, 1 mm l-ascorbate, and 50 μm Fe(NH_4_)_2_(SO_4_)_2_ or 4 μm PHD2, 100 μm substrate, 300 μm 2OG, 4 mm l-ascorbate, and 50 μm Fe(NH_4_)_2_(SO_4_)_2_. For HIF-1α CODD_19-mer_, the background levels of methionine oxidation resulting from assay conditions were subtracted based on non-enzymatic controls (48). Determination of initial velocities in steady-state kinetic experiments was performed varying the (co-)substrate concentrations and maintaining concentrations of components at levels higher than 2 × *K*_*m*_^app^ to ensure saturation conditions.

The O_2_ dependence of FIH-catalyzed reactions was determined by conducting enzymatic assays in an “*In vivo* 500 Hypoxia Workstation” (Ruskinn) using buffers pre-equilibrated under different pO_2_ values for 24 h. The O_2_ dependence of PHD2-catalyzed reactions was determined by conducting enzymatic assays in sealed glass vials with variable N_2_/O_2_ contents (42). Reactions were stopped by spotting samples onto a matrix-assisted laser desorption/ionization-time of flight mass spectrometry (MALDI-TOF-MS) target plate and immediately mixing with α-cyano-4-hydroxycinnamic acid matrix. Hydroxylation levels were assessed by MALDI-TOF-MS as described (46).

##### Stopped-flow UV-visible Spectroscopy Experiments

Stopped-flow UV-visible spectroscopy experiments were performed as described previously (32, 42). Anerobically prepared assay mixtures containing 0.5 mm apo-FIH, 1 mm substrate, 5 mm 2OG, 0.4 mm Fe(II) or 0.8 mm apo-PHD2, 1 mm substrate, 5 mm 2OG, 0.5 mm Fe(II) were rapidly mixed with O_2_-free/O_2_-saturated buffer. The reaction was observed over 1000 s using a photodiode array detector, and kinetic traces were analyzed using Origin version 8.51 software. Data were fitted with a double exponential function (*y* = *A*_1_ × exp(−*k*_rise_*t*) + *A*_2_ × exp(−*k*_fall_*t*) + *y*_0_). The *A*_1_, *A*_2_, and *y*_0_ parameters were restricted with upper limits to match the observed amplitude/offset changes.

##### Rapid Quench-Flow Experiments

Rapid quench-flow experiments were performed using the same conditions and preparation of the assay mixture as used in stopped-flow experiments. Rapid quench-flow equipment (TgK Scientific Ltd.) in an anaerobic glove box was used for the experiments. Samples were quenched with 1% CF_3_CO_2_H and analyzed using MALDI-TOF-MS (peptide hydroxylation) as described above or liquid chromatography-mass spectrometry (LC-MS, for succinate production). For LC-MS, chromatographic separation was performed at 50 °C using a Waters ACQUITY BEH Amide 1.7-μm, 2.1 × 100-mm column on a Waters ACQUITY^TM^ ultraperformance liquid chromatography system (Waters Corp., Milford, MA). The following eluents were used: mobile phase A: 10% H_2_O, 90% acetonitrile (v/v), 10 mm ammonium formate; mobile phase B: 50% H_2_O, 50% acetonitrile (v/v), 10 mm ammonium formate. The elution gradient was 0–7.0 min linear from 10 to 30% B, and 7.0–9.0 min at 10% B for re-equilibration of the column. A constant flow rate of 0.4 ml/min was used. Analytes were detected in negative ionization mode using single reaction monitoring on a Quattro triple quadrupole mass spectrometer (Waters) with a cone voltage of 15 V and a capillary voltage of 3.0 kV. The desolvation temperature was set to 250 °C, and the source temperature was set to 120 °C.

##### Intact protein LC-MS

Samples obtained from FIH kinetic assays with 2CA and 3CA were diluted to a substrate concentration of 5 μm and then analyzed using a Waters® LCT Classic mass spectrometer by electrospray ionization in positive ion mode. An Agilent HP1100 LC system was used, and samples were run through a Phenomenex® Aeris Widepore 3.6-μm XB-C8 150 × 2.1-mm column at 0.5 ml/min in a gradient of phase A (95% H_2_O, 5% CH_3_CN, 0.01% formic acid) to phase B (95% CH_3_CN, 5% H_2_O, 0.01% formic acid). The LC/MS used the following method: 0–1.5 min, 100% A and then 1.5–12 min linear gradient from 0 to 100% B, 12–14 min 100% B, and 15.0–19.0 min 100% A for re-equilibration of the column. Data were collected and analyzed using MassLynx version 4.0.

##### CD Spectroscopy

CD spectra were recorded using a Chirascan CD/fluorimeter (Applied Photophysics) spectrometer. Measurements were carried out at 20 °C in a 0.1 cm path length quartz cuvette, with protein concentration of 0.125 mg/ml in 5 mm sodium phosphate buffer (pH 8.0). Spectra were recorded in the wavelength range of 260–190 nm at 0.5-nm intervals, and each spectrum represents an average of three scans. Spectra were baseline-corrected by subtraction of the buffer spectrum. Data were expressed as the mean residue ellipticity ([θ], degrees·cm^2^·dmol^−1^). Thermal denaturation experiments were performed under the same conditions. The ellipticity at 220 nm (θ_220_) was monitored from 10 to 90 °C.

##### MALDI-TOF/TOF-MS/MS

**FIGURE 2.**
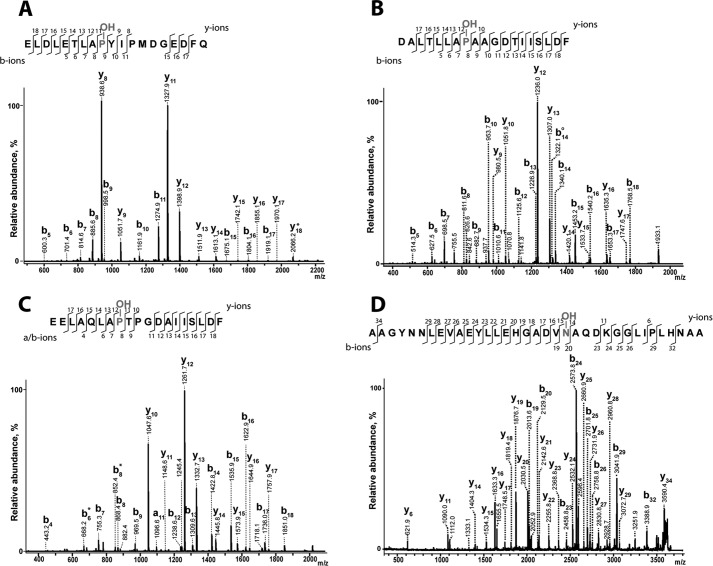
**Assignment of modification sites by MALDI-TOF/TOF laser-induced dissociation fragmentation of hydroxylated peptide substrates.** Modification sites were confirmed by enzymatic hydroxylation of synthetic peptide substrates followed by MS/MS analysis with MALDI-TOF/TOF laser-induced dissociation. *A*, CODD HIF-2α. *B*, NODD HIF-2α. *C*, NODD HIF-1α. *D*, tankyrase-1 (35-mer). Assay conditions were as follows. 4–5 μm enzyme, 100 μm peptide, 50 μm Fe(II), 300 μm 2OG, 1 mm l-ascorbate in HEPES 50 mm (pH 7.5) were incubated at 37 °C at atmospheric O_2_ for 20 min.

Hydroxylation sites were assigned unambiguously by MALDI-TOF/TOF mass spectrometry (Fig. 2) using a Bruker Ultraflex instrument, essentially as described previously (25). Thus, peptide samples were spotted onto an AnchorChip target plate with an α-cyano-4-hydroxycinnamic acid matrix using the dried droplet method. MS and MS/MS spectra were acquired by manual operation in reflectron mode, and the instrument was calibrated directly prior to data acquisition using monoisotopic peptide masses with Peptide Calibration Standard II (Bruker Daltonics, Coventry, UK). Sample ionization was achieved with a nitrogen laser (337 nm), and MS/MS spectra were generated by laser-induced dissociation (49). Assignment of hydroxylation sites in other peptides used in this study was performed previously (8, 21, 22, 26, 50).

## Results

### 

#### 

##### Steady-state Kinetic Studies of FIH- and PHD2-catalyzed Hydroxylation of HIF-1/2α

**FIGURE 3.**
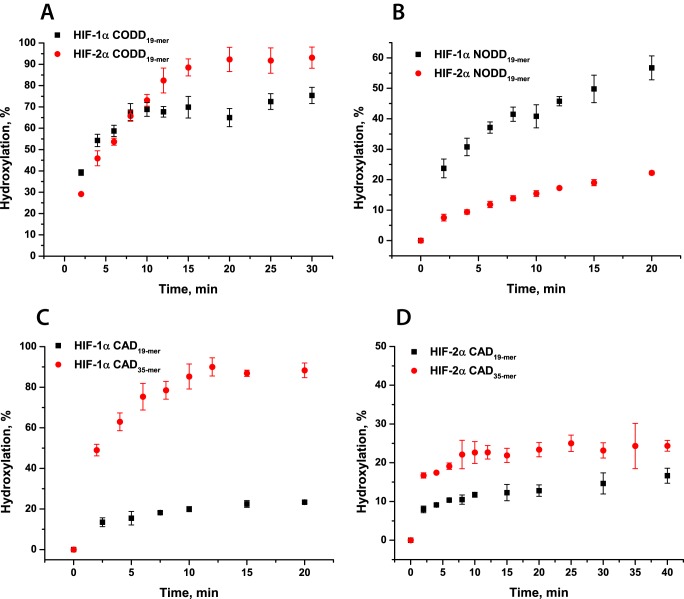
**PHD2- and FIH-catalyzed hydroxylation of HIF-1/2α peptide substrates.** Sequences of the peptides are given in Table 1. *A*, time course of HIF-1/2α CODD_19-mer_ peptide hydroxylation; *B*, time course of HIF-1/2α NODD_19-mer_ peptide hydroxylation. Conditions were as follows: 4 μm PHD2, 100 μm peptide, 50 μm Fe(II), 300 μm 2OG, 1 mm l-ascorbate in HEPES 50 mm (pH 7.5) were incubated at 37 °C at atmospheric O_2_. *C*, time course of HIF-1α CAD 19- and 35-mer peptide hydroxylation. *D*, time course of HIF-2α CAD 19- and 35-mer peptide hydroxylation. Conditions were as follows: 5 μm FIH, 500 μm peptide, 50 μm Fe(II), 500 μm 2OG, 1 mm l-ascorbate in HEPES 50 mm (pH 7.5) were incubated at 37 °C at atmospheric O_2_. The different hydroxylation levels were analyzed by MALDI-TOF-MS. *Error bars*, S.D. of triplicate assays.

As a prelude to studies on the reaction of PHD2 and FIH with O_2_, (apparent) *K_m_* values were determined for 2OG and HIF peptide substrates (HIF-1/2α CODD/NODD/CAD; Table 1) under standard conditions. Enzyme assays were conducted as described under “Experimental Procedures,” and levels of peptide hydroxylation were analyzed using MALDI-TOF-MS. Differences were observed in the relative efficiencies of catalysis in overall time course assays; consistent with a previous report (46), PHD2 showed a higher level of activity toward CODD (70–90% substrate hydroxylation after 15 min for both HIF-1α and HIF-2α, respectively) than NODD (50 and 20% substrate hydroxylation for HIF-1α and HIF-2α, respectively) (Fig. 3, *A* and *B*). Under the standard conditions, FIH only hydroxylated 10–20% of HIF-α 19-mer fragments; therefore, for further studies, 35-mer HIF-α CAD peptides were used. Requirements of longer peptides to observe more efficient FIH-catalyzed hydroxylation has been reported and is likely related to insufficient binding of short CAD peptides (10, 40, 51). Increasing the length of the HIF-1α CAD peptide resulted in nearly complete substrate hydroxylation, whereas analyses increasing the length of the HIF-2α CAD showed only a 10% increase in activity and <30% substrate hydroxylation (Fig. 3, *C* and *D*).

**TABLE 1:** **Sequences of the peptide substrates used in this study** Where peptides represent fragments of a protein, amino acid numbers are given in parentheses.

Peptide	Sequence
CODD HIF-1α_19-mer_ (556–574)	DLDLEMLAPYIPMDDDFQL
CODD HIF-2α_19-mer_ (523–541)	ELDLETLAPYIPMDGEDFQ
NODD HIF-1α_19-mer_ (395–413)	DALTLLAPAAGDTIISLDF
NODD HIF-2α_19-mer_ (398–416)	EELAQLAPTPGDAIISLDF
CAD HIF-1α_19-mer_ (789–806)	DESGLPQLTSYDCEVNAPI
CAD HIF-1α_35-mer_ (789–822)	DESGLPQLTSYDCEVNAPIQGSRNLLQGEELLRAL
CAD HIF-2α_19-mer_ (832–850)	ESYLLPELTRYDCEVNVPV
CAD HIF-2α_35-mer_ (832–866)	ESYLLPELTRYDCEVNVPVLGSSTLLQGGDLLRAL
1CA_20-mer_	HLEVVKLLLEAGADVNAQDK
tankyrase-1_20-mer_ (849–868)	NLEVAEYLLEHGADVNAQDK
tankyrase-1_35-mer_ (844–886)	AAGYNNLEVAEYLLEHGADVNAQDKGGLIPLHNAA
2CA	SDKNGSTPLHLAARNGHLEVVKLLLEHGADVNAQDKWGKTAFDISIDNGNEDLAEILQ
3CA	GSHMGSDLGKKLLEAARAGQDDEVRILMANGADVAAKDKNGSTPLHLAARNGHLEVVKLLLEAGADVNAQDKFGKTAFDISIDNGNEDLAEILQ

Steady-state kinetic parameters were then determined for PHD2 and FIH. Consistent with previous reports (11, 40, 46), PHD2 has a higher affinity for 2OG than FIH (*K*_*m*_^app^(2OG) = 13 ± 2 μm for PHD2 with CODD HIF-1α compared with 110 ± 20 μm for FIH with HIF-1α CAD_35-mer_), and *k*_cat_^app^ values revealed more efficient PHD2-catalyzed hydroxylation of CODD than NODD substrates with both HIF-1α and HIF-2α (*e.g.* 0.060 ± 0.006 and 0.028 ± 0.001 s^−1^ for CODD and NODD HIF-1α, respectively, Table 2; kinetic constants derived from data in supplemental Figs. S1 and S2). The determined parameters for FIH were consistent with the reported more efficient FIH-catalyzed hydroxylation of HIF-1α compared with HIF-2α CAD (Table 2; kinetic constants derived from data in supplemental Fig. S3) (10, 40). Substrate inhibition was observed at high 2OG concentrations (>100 μm for HIF-1α CAD and >700 μm for HIF-2α CAD peptide; Table 2).

**TABLE 2:** **Steady-state kinetic parameters of FIH and PHD2 catalysis** Conditions were as follows. 0.1–5 μm FIH, peptide substrate (varied), 50 μm Fe(II), 2OG (varied), and 1 mm l-ascorbate in 50 mm HEPES (pH 7.5) were incubated at 37 °C. 4 μm PHD2, peptide substrate (varied), 50 μm Fe(II), 2OG (varied), and 4 mm l-ascorbate in 50 mm HEPES (pH 7.5) were incubated at 37 °C. Hydroxylation levels were analyzed by MALDI-TOF-MS. The dependences of the initial rate on the substrate concentration are presented in supplemental Figs. S1–S3.

Enzyme	Substrate	*K*_*m*_^app^(2OG)	*K*_*m*_^app^(peptide)	*K*_*m*_^app^(O_2_)	*k*_cat_^app^
		μ*m*	μ*m*	μ*m*	*s*^−*1*^
FIH	HIF-1α CAD_35-mer_	110 ± 20	180 ± 30	110 ± 30	0.56 ± 0.04
FIH	HIF-2α CAD_35-mer_	19 ± 6*^a^*	315 ± 40*^a^*	110 ± 10	0.049 ± 0.005
PHD2	HIF-1α CODD	13 ± 2	10 ± 6	460 ± 30	0.060 ± 0.006
PHD2	HIF-1α NODD	30 ± 9	11 ± 2	>450	0.028 ± 0.001
PHD2	HIF-2α CODD	9 ± 2	34 ± 10	>450	0.069 ± 0.006
PHD2	HIF-2α NODD	17 ± 5	50 ± 8	410 ± 80	0.013 ± 0.001

*^a^* Substrate inhibition at 2OG concentrations >100 μm and HIF-2α CAD_35-mer_ concentrations >700 μm was observed.

**FIGURE 4.**
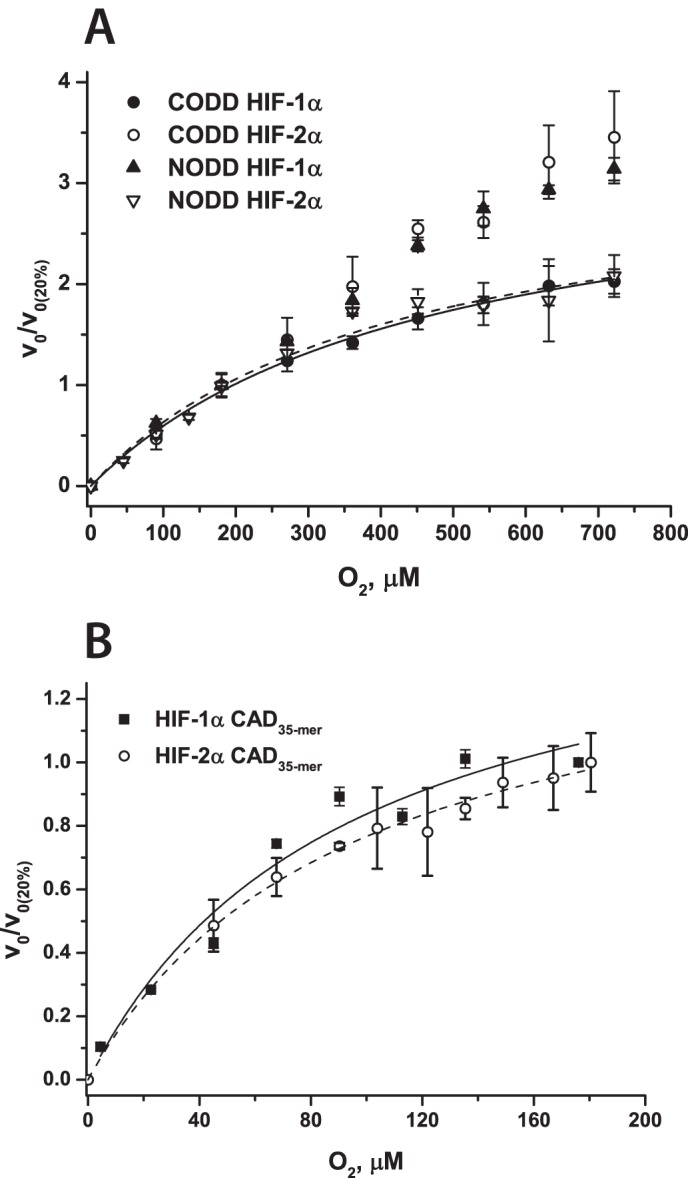
**O_2_ dependence of FIH- and PHD2-catalyzed hydroxylation of HIF-1/2α.** *A*, O_2_ dependence of PHD2-catalyzed hydroxylation of HIF-1/2α CODD/NODD 19-mer peptides. 4 μm PHD2, peptide (varied), 50 μm Fe(II), 2OG (varied), 4 mm l-ascorbate in HEPES 50 mm (pH 7.5) were incubated at 37 °C. The different O_2_ levels were maintained using mass flow controllers (42), and the hydroxylation levels were analyzed by MALDI-TOF-MS. *B*, O_2_ dependence of FIH-catalyzed hydroxylation of CAD HIF-1/2α 35-mer peptides. Conditions were as follows. 5 μm FIH, 500 μm peptide, 50 μm Fe(II), 1 mm 2OG, 1 mm l-ascorbate in HEPES 50 mm (pH 7.5) were incubated at 37 °C in a hypoxia work station under different *p*O_2_. To compare reactions with different *V*_max_, the relative initial rates (to the initial rate of the reaction at 20% O_2_) are presented. *Error bars*, S.D. of triplicate assays.

To subsequently study the O_2_ dependence of the PHD2- and FIH-catalyzed reaction, the initial rates of hydroxylation were determined either in a hypoxic work station or by performing the reaction in sealed glass vials under N_2_/O_2_ gas mixes with variable O_2_ concentration (42). Half-saturation of O_2_ for FIH-catalyzed hydroxylation of HIF-1α CAD peptides was achieved at 9–11% O_2_, giving *K*_*m*_^app^(O_2_) as 110 ± 30 μm, which was much lower than for PHD2-catalyzed hydroxylation of CODD/NODD HIF-1/2α (*K*_*m*_^app^(O_2_) > 400 μm in all cases), consistent with previous reports (Fig. 4) (10, 11, 42). Notably, these results are consistent with previous *K*_*m*_^app^(O_2_) estimates for FIH and are also supportive of the reported ability of FIH to retain activity to a greater degree of hypoxia than the PHDs in cells (39). Interestingly, despite the difference in final hydroxylation levels and steady-state product accumulation time courses, there was no difference in *K*_*m*_^app^(O_2_) for HIF-1α CAD 19- and 35-mer peptides, suggesting no influence of substrate binding on the affinities of O_2_ to an enzyme·substrate complex when saturating concentrations of the peptides are used (supplemental Fig. S4).

##### Pre-steady-state Kinetic Studies of the Reactions of PHD2·Fe(II)·2OG·HIF-α CODD/NODD Substrates with O_2_

Previously reported pre-steady-state kinetic studies on PHD2 focused on its reaction with CODD HIF-1α 19-mer peptide (38, 42); there is no reported information on the rates of PHD2 reaction with O_2_ in the presence of NODD HIF-1α or HIF-2α substrates. To ascertain whether a slow reaction with O_2_ under pre-steady-state conditions is common to PHD2-catalyzed hydroxylation of both CODD and NODD, we investigated the reaction of PHD2 with NODD substrates in comparison with CODD for both HIF-1α and HIF-2α. Pre-steady-state reactions of PHD2 were initiated by rapid mixing of anaerobic PHD2·Fe(II)·2OG·HIF-α complexes with O_2_-saturated buffer at 5 °C. Reactions were initially observed using a photodiode array detector, and difference spectra (in comparison with the reaction with O_2_-free buffer) were subsequently analyzed.

**FIGURE 5.**
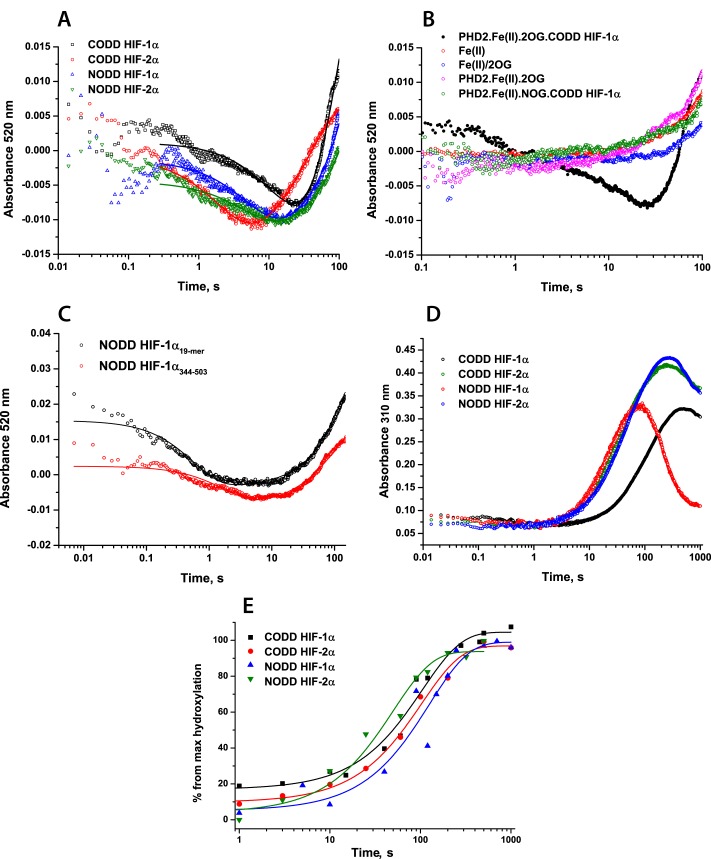
**Pre-steady-state kinetics of PHD2-catalyzed HIF-1/2α hydroxylation.** Reaction mixtures containing anaerobic 0.8 mm apo-PHD2, 0.5 mm Fe(II), 5 mm 2OG, 1 mm peptide (19-mer), or protein substrate in HEPES 50 mm (pH 7.5) were rapidly mixed with O_2_-saturated buffer at 5 °C in a 1:1 ratio. The sequences of the peptides are given in Table 1. *A*, kinetic traces at λ = 520 nm showing O_2_-dependent consumption of the initial PHD2·Fe(II)·2OG·peptide complexes. *B*, stopped-flow kinetic traces at 520 nm showing absorbance changes in control experiments: Fe(II) only, mix of Fe(II) and 2OG, reaction of PHD2·Fe(II)·2OG complex with O_2_ in the absence of CODD; reaction of PHD2·Fe(II)·*N*-oxalylglycine (*NOG*)·CODD complex with O_2_. *N*-Oxalylglycine is an inhibitor of 2OG oxygenases. *C*, stopped-flow kinetic traces at 520 nm showing absorbance species formed on PHD2-catalyzed HIF-1α NODD 19-mer peptide hydroxylation (*black*) and recombinant protein (HIF-1α(344–503)) hydroxylation (*red*). *D*, stopped-flow kinetic traces at 310 nm showing absorbance species formed in the PHD2-catalyzed HIF-1/2α hydroxylation. The kinetic traces represent the average of triplicate assays. *E*, HIF-1/2α peptide hydroxylation, as determined by MALDI-TOF-MS. Reactions were quenched with 1% CF_3_CO_2_H at defined time points.

The formation of an enzyme·substrate complex for 2OG oxygenases is characterized by a weak absorbance feature at 520–530 nm due to metal ligand charge transfer interactions of Fe(II) and 2OG (32–34, 38, 53). Consistent with the consumption of the enzyme·substrate complex, and dependent on active PHD2·Fe(II)·2OG·substrate complex, a decay in absorbance at 520 nm was observed during the PHD2-catalyzed reaction, with the minimum reached at ∼20 s, after which an absorbance increase was observed (Fig. 5, *A* and *B*). Although this absorbance increase is consistent with reformation of an enzyme·substrate complex (32) (PHD2·Fe(II)·2OG and PHD2·Fe(II)·2OG·substrate formation would both give rise to absorbance increases in this region), calculated rates are probably also influenced by off-pathway processes (*e.g.* accumulation of Fe(III)) (42). Thus, we analyzed rates of 520 nm absorbance decay for PHD2-catalyzed CODD HIF-1α hydroxylation and other peptide substrates (CODD/NODD HIF-1/2α) (Fig. 5*A* and Table 3). Comparison of kinetics in the presence of CODD HIF-1α and HIF-2α peptides revealed faster O_2_-initiated decay of this species in the case of HIF-2α (0.040 ± 0.007 and 0.450 ± 0.010 s^−1^ for CODD HIF-1α and HIF-2α, respectively). Equivalent studies of complexes in the presence of NODD HIF-1α and HIF-2α revealed nearly equivalent rates of 0.178 ± 0.002 and 0.195 ± 0.003 s^−1^, respectively.

**TABLE 3:** **Kinetic parameters of pre-steady-state PHD2- and FIH-catalyzed hydroxylation** Conditions were as follows: an anaerobically prepared mixture of 0.8 mm PHD2, 1 mm peptide substrate, 5 mm 2OG, and 0.7 mm Fe(II) or 0.5 mm FIH, 1 mm peptide substrate, 5 mm 2OG, and 0.4 mm Fe(II) in 50 mm HEPES (pH 7.5) was rapidly mixed with O_2_-saturated buffer at 5 °C. ND, not determined.

Enzyme	Substrate	Stopped-flow, 520 nm (PHD2), 500 nm (FIH)		Rapid quench-flow	
		*k*_fall phase_	*k*_rise phase_	*k*_product_	*k*_succinate_
		*s*^−*1*^	*s*^−*1*^	*s*^−*1*^	*s*^−*1*^
PHD2	CODD HIF-1α	0.040 ± 0.007	0.015 ± 0.005	0.0155 ± 0.0012	0.0130 ± 0.0020
PHD2	CODD HIF-2α	0.450 ± 0.010	0.027 ± 0.001	0.0124 ± 0.0030	0.0120 ± 0.0070
PHD2	NODD HIF-1α	0.178 ± 0.006	0.002 ± 0.001	0.0080 ± 0.0020	0.0060 ± 0.0010
PHD2	NODD HIF-2α	0.195 ± 0.009	0.003 ± 0.001	0.0199 ± 0.0032	0.0270 ± 0.0060
FIH	CAD HIF-1α_35-mer_	4.5 ± 0.3	0.009 ± 0.005	0.33 ± 0.07	0.3 ± 0.1
FIH	CAD HIF-2α_35-mer_	4.9 ± 0.2	0.017 ± 0.01	0.12 ± 0.01	0.11 ± 0.03
FIH	CAD HIF-1α_19-mer_	ND	ND	0.013 ± 0.003	0.016 ± 0.004

To investigate the rate of the PHD-catalyzed reaction with longer HIF substrates, equivalent experiments were conducted with recombinant HIF-1α(344–503) NODD protein; these showed the same kinetics of PHD2·Fe(II)·2OG·NODD complex consumption as was observed in the presence of the 19-mer peptide (Fig. 5*C*), confirming that peptide binding is not rate-limiting. Consistent with a previous report (42), the formation and degradation of a transient species absorbing at λ_max_ = 310 nm was observed (in this and other pre-steady-state assays in this study; Fig. 5*D*); however, the slow rates of its formation suggest that (consistent with our previous observations (38, 42)) an Fe(IV)-oxo intermediate (λ_max_ ∼320 nm (32)) species probably does not accumulate during PHD2 catalysis.

To investigate correlation of the spectroscopic observations with rates of product accumulation, equivalent reactions were analyzed by rapid quench-flow methodology. Anerobically prepared PHD2·Fe(II)·2OG·peptide substrate complexes were mixed with O_2_-saturated buffer in a 1:1 ratio; reactions were quenched at defined time points with 1% aqueous CF_3_CO_2_H. The level of peptide hydroxylation was assessed by MALDI-TOF-MS (42), and succinate accumulation was analyzed by LC-MS. To obtain rate constants for product accumulation, the results were fitted with a single exponential function (Fig. 5*E* and Table 3). Besides NODD HIF-2α being hydroxylated at a faster rate than NODD HIF-1α, our results showed no significant difference in the kinetics of hydroxylated product formation for PHD2 in the presence of any of the tested NODD and CODD substrates. These results suggest that the more rapid enzyme·substrate complex depletion observed for CODD HIF-2α did not affect the rate-limiting step in product formation. The time courses of succinate accumulation (reflecting 2OG turnover) were coupled to peptide hydroxylation within the limits of detection (Table 3 and supplemental Fig. S5).

##### Pre-steady-state Kinetic Studies of the Reactions of FIH·Fe(II)·2OG·HIF-1/2α CAD Substrates with O_2_

Although the pre-steady-state reaction of PHD2 with O_2_ has been previously investigated (38, 42), to date, no studies on the pre-steady-state kinetics of FIH have been reported. We carried out stopped-flow and rapid quench-flow experiments to compare the reaction of FIH with O_2_ with that of PHD2 and to relate the results to the differences reported in *K*_*m*_^app^(O_2_) values.

**FIGURE 6.**
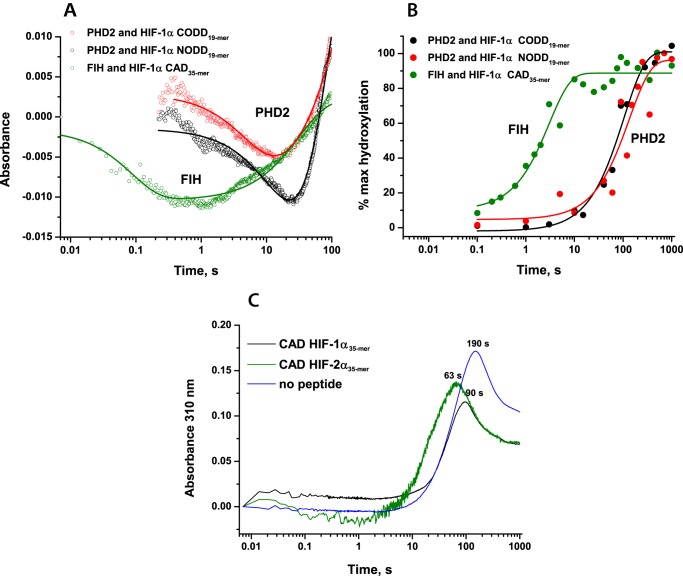
**Comparison of the O_2_-initiated PHD2- and FIH-catalyzed HIF-α hydroxylation reactions under pre-steady-state conditions.** Reaction mixtures containing anaerobic 0.8 mm apo-PHD2, 0.5 mm Fe(II), 5 mm 2OG, 1 mm peptide or 0.5 mm FIH, 0.4 mm Fe(II), 5 mm 2OG, 1 mm peptide in HEPES 50 mm (pH 7.5) were rapidly mixed with O_2_-saturated buffer at 5 °C in a 1:1 ratio. *A*, kinetic traces at λ = 520 nm (for PHD2) or λ = 500 nm (for FIH) showing O_2_-dependent consumption of the initial enzyme·Fe(II)·2OG·peptide complexes. The kinetic traces represent the average of triplicate assays. *B*, FIH- and PHD2-catalyzed HIF-1α peptide hydroxylation under the same conditions, as determined by MALDI-TOF-MS. Reactions were quenched with 1% CF_3_CO_2_H at defined time points. *C*, formation and degradation of 310 nm species in the FIH-catalyzed HIF-1/2α CAD_35-mer_ hydroxylation.

Upon rapid mixing of anaerobic FIH·Fe(II)·2OG·HIF-α complexes with O_2_-saturated buffer at 5 °C, a decay in absorbance in the 500 nm region and subsequent increase resulting in a minimum at ∼0.3–3 s was observed in stopped-flow UV-visible absorbance experiments, as reported for PHD2 and other 2OG oxygenases (Fig. 6*A* and Table 3) (32–34, 36, 53). Kinetic traces were fitted using a double exponential function; the fitting parameters of intermediate formation and decay are summarized in Table 3. The results reveal that the 500 nm decay occurs much more rapidly for FIH compared with PHD2: 4.5 ± 0.3 s^−1^ for FIH with CAD HIF-1α_35-mer_ and 0.040 ± 0.007 s^−1^ for PHD2 with CODD HIF-1α, suggesting a faster O_2_-initiated reaction for FIH (Fig. 6*A*).

The kinetic constant of product formation for the FIH·Fe(II)·2OG·HIF-1α CAD_35-mer_ complex upon reaction with O_2_ was determined to be 0.33 ± 0.07 s^−1^ (Table 3 and Fig. 6*B*). This was faster than the kinetic constant of product formation for an FIH·Fe(II)·2OG·HIF-2α CAD_35-mer_ complex upon reaction with O_2_ (0.12 ± 0.01 s^−1^ (Table 3 and supplemental Fig. S6). A lower *k*_cat_^app^ value was also observed in steady-state experiments (0.56 ± 0.04 and 0.049 ± 0.005 s^−1^ for HIF-1α and HIF-2α, respectively; Table 2 and supplemental Fig. S3). Notably, the O_2_-initiated rates of product accumulation were significantly faster in the case of FIH compared with PHD2 (Fig. 6*B*; rate constants, 0.33 ± 0.07 s^−1^ for FIH with CAD HIF-1α_35-mer_ and 0.0155 ± 0.0012 s^−1^ for PHD2 with CODD HIF-1α), consistent with the differences in 500 nm decay rates observed by stopped-flow analyses and potentially related to the reduced *K*_*m*_^app^(O_2_) values for FIH compared with PHD2.

During the stopped-flow analyses, we observed late formation of a transient species with maximum absorbance at 310 nm and a maximum accumulation time at 50–100 s, as observed for PHD2 (Fig. 6*C*). Comparison with the O_2_-activated product formation studied by rapid quench-flow experiments revealed that this species is formed after the hydroxylation of the peptide substrates was complete, suggesting that a detectable Fe(IV)-oxo intermediate does not accumulate during the FIH-catalyzed reaction. The observed accumulation of a 310-nm transient species can be rationalized by the formation of a transient iron species as a result of substrate-uncoupled 2OG turnover subsequent to consumption of all prime substrate and/or an oxidation of bound or unbound Fe(II) (42). Thus, similar to the observations for PHD2 (38, 42), accumulation of an Fe(IV)-oxo intermediate was not observed (at 320 nm) in FIH catalysis, at least with our tested substrates.

##### Steady-state Kinetic Studies of FIH-catalyzed Hydroxylation of ARD Substrates

FIH catalyzes hydroxylation of multiple ARD substrates (18, 19, 21, 23, 43, 44). It has been reported that the FIH *K*_*m*_^app^(O_2_) value for ARD-containing proteins Notch1–3 is 10-fold lower than for HIF-1α (43), suggesting a relatively higher affinity of FIH for O_2_ when catalyzing ARD hydroxylation, compared with HIF-α hydroxylation. We therefore wished to further investigate the kinetic dependence of FIH on O_2_ with respect to ARD and HIF substrates. We compared FIH hydroxylation of a consensus ankyrin repeat 20-mer peptide (1CA_20-mer_, designed based on ARD sequence analyses (21, 54, 55)) and a 20-mer fragment of tankyrase-1, tnkrs-1_20-mer_ (a multifunctional poly(ADP-ribose) polymerase containing 24 ARDs) to HIF-α isoforms by determining steady-state kinetic parameters for these substrates as a prelude to investigating O_2_ dependence (43). Overall, the *K*_*m*_^app^ values for ankyrin repeat peptide FIH substrates are all lower than for either HIF-1α or HIF-2α CAD peptide substrates (∼180–300 μm for HIF-1/2α CAD and <100 μm for ankyrin substrates (Table 4); kinetic constants derived from data in supplemental Fig. S7), supporting the previously reported stronger binding of ARDs to FIH (43). At the same time, *k*_cat_^app^ values for ankyrin peptides were higher than for HIF-α (1.6 ± 0.1 and 0.56 ± 0.04 s^−1^ for 1CA_20-mer_ and HIF-1α CAD_35-mer_, respectively), implying more efficient turnover of the ankyrin substrates (Table 4 and supplemental Fig. S7), again consistent with previous reports (43).

**TABLE 4:** **Steady-state kinetic parameters of FIH-catalyzed ARD hydroxylation** Conditions were as follows: 0.1–5 μm FIH, substrate (varied), 50 μm Fe(II), 2OG (varied), and 1 mm l-ascorbate in 50 mm HEPES (pH 7.5) were incubated at 37 °C. Hydroxylation levels were analyzed by MALDI-TOF-MS or LC-MS. The dependences of the initial rate on the substrate concentration are presented in supplemental Figs. S7 and S8. ND, not determined.

Enzyme	Substrate	*K*_*m*_^app^(2OG)	*K*_*m*_^app^(peptide)	*K*_*m*_^app^(O_2_)	*k*_cat_^app^
		μ*m*	μ*m*	μ*m*	*s*^−*1*^
FIH	1CA_20-mer_	290 ± 50	25 ± 7	40 ± 10	1.6 ± 0.1
FIH	tnkrs-1_20-mer_	370 ± 120	78 ± 11	ND	0.8 ± 0.1
FIH	tnkrs-1_35-mer_	61 ± 7	14 ± 4	50 ± 10	3.0 ± 0.2
FIH	2CA	110 ± 30	16 ± 3	56 ± 15	1.7 ± 0.2
FIH	3CA	200 ± 60	60 ± 15	120 ± 20	0.6 ± 0.2

**FIGURE 7.**
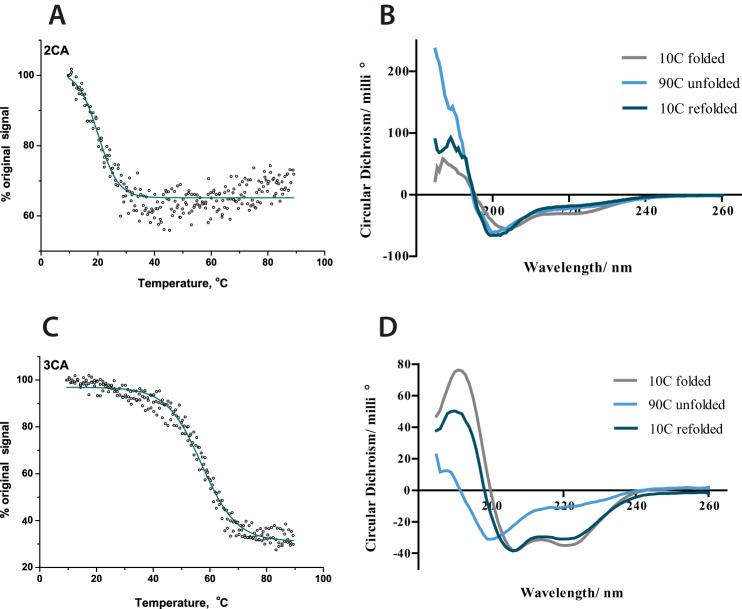
**CD spectroscopic comparison of 2CA and 3CA secondary structure.** Thermal denaturation experiments of 2CA and 3CA are shown. Conditions were as follows. 0.2 mg/ml protein in 10 mm sodium phosphate buffer (pH 8.0) was studied at 222 nm for thermal denaturation experiments between 10 and 90 °C. *A*, melting curves of 2CA. *B*, spectra of 2CA between 260 and 190 nm at 10 °C, after heating to 90 °C and after cooling again to 10 °C. *C*, melting curves of 3CA. *D*, spectra of 3CA between 260 and 190 nm at 10 °C, after heating to 90 °C, where the α-helical structure is predominantly lost, and after cooling again to 10 °C, where the structure is largely regained.

ARD proteins probably must undergo at least partial unfolding from their consensus fold prior to productive FIH-catalyzed hydroxylation (21, 44). To investigate the effect of ankyrin repeat folding on the efficiency of hydroxylation, 1CA_20-mer_ was compared with two- and three-consensus ankyrin repeat proteins (2CA and 3CA). The CA repeat sequence was designed previously based on bioinformatics studies of multiple natural ARD sequences (21, 54, 55). Circular dichroism experiments showed that whereas 2CA does not possess significant secondary structure (2CA is unfolded at >10 °C), 3CA is folded in solution (Fig. 7 and Refs. 21, 22). The kinetic parameters for 1CA_20-mer_, 2CA, and 3CA were determined. They showed a decrease in substrate and co-substrate *K*_*m*_^app^ values for 2CA compared with 1CA_20-mer_ (*K*_*m*_^app^(substrate) = 25 ± 7 and 16 ± 3 μm, for 1CA and 2CA, respectively) in line with increased efficiency of longer substrates for FIH but an increase in the *K*_*m*_^app^ value for the 3CA substrate (60 ± 15 μm (Table 4); kinetic constants derived from data in supplemental Fig. S8). Notably, there was a reduction in turnover rate for 3CA substrate. These results suggest that the requirement for at least partial unfolding of the ankyrin repeat prior to FIH-catalyzed hydroxylation affects catalytic efficiency (21, 44).

**FIGURE 8.**
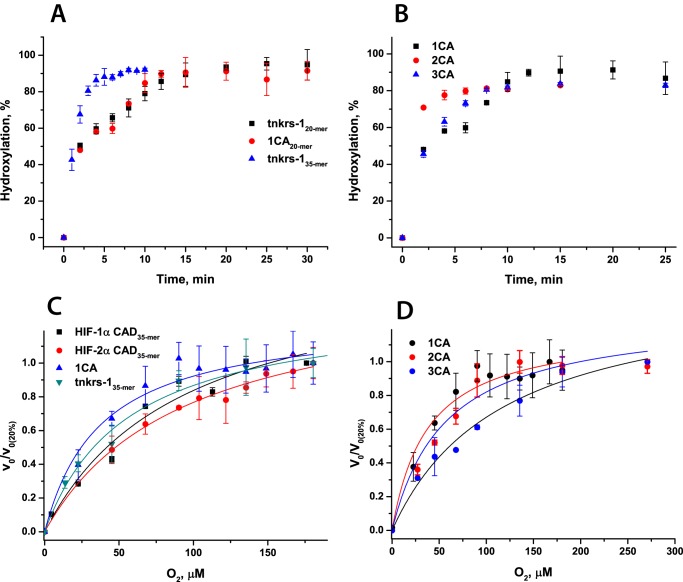
**Time course and O_2_ dependence of the FIH-catalyzed hydroxylation of HIF-α CAD and ARD substrates.** Sequences of the peptides are given in Table 1. Conditions were as follows. 5 μm FIH, 500 μm peptide, 50 μm Fe(II), 1 mm 2OG, 1 mm l-ascorbate in HEPES 50 mm (pH 7.5) were incubated at 37 °C. For O_2_ dependence studies, reactions were carried out in a hypoxic work station under different *p*O_2_ (assays at >20% O_2_ conducted using mass flow controllers (42)). The different hydroxylation levels were analyzed by MALDI-TOF-MS or by LC-MS (for 2CA and 3CA). To compare reactions with different *V*_max_, the relative initial rates (relative to the initial rate of the reaction at 20% O_2_) are presented. *Error bars*, S.D. of triplicate assays. *A*, time course of consensus ARD substrate hydroxylation (tankyrase-1 (tnkrs-1 20-mer and 35-mer) and one-consensus ankyrin repeat 20-mer (1CA)). *B*, time course of ARD peptide hydroxylation (one-consensus ankyrin repeat 20-mer (1CA), two ankyrin repeats (2CA), and three ankyrin repeats (3CA)). *C*, O_2_ dependence of HIF-1/2α CAD, 1CA, and tnkrs-1_35-mer_ hydroxylation. *D*, O_2_ dependence of 1CA, 2CA, and 3CA hydroxylation.

We then determined and compared *K*_*m*_^app^(O_2_) values for our ankyrin substrates with those determined for HIF-1/2α CAD (Table 4 and Fig. 8). The *K*_*m*_^app^ (O_2_) values for 1CA and tnkrs-1_35-mer_ were lower than those determined for HIF substrates (40 ± 10, 50 ± 10, and 110 ± 30 μm for 1CA, tnkrs-1_35-mer_, and HIF-1α_35-mer_, respectively; note that the more efficient 35-mer tnkrs-1 peptide was used to compare with the equivalent length HIF-1α_35-mer_). These results are supportive of the reported comparison of Notch1–3 *K*_*m*_^app^(O_2_) values (43), strengthening the proposal that binding of different protein substrates can modulate the reaction/affinity of FIH for O_2_. There was little difference in the FIH affinities to O_2_ in the presence of the 1CA and 2CA substrates as determined by *K*_*m*_^app^(O_2_) values (40 ± 10 and 56 ± 15 μm for 1CA and 2CA, respectively). Compared with the substantially unfolded 1CA or 2CA, however, the *K*_*m*_^app^(O_2_) in the presence of 3CA (120 ± 20 μm) was 2-fold higher than the values in the presence of 1CA and 2CA and similar to that in the presence of HIF-α (110 ± 30 μm). Thus, increasing the number of ankyrin repeats and folding of the 3CA does not appear to facilitate the reaction of FIH with O_2_; this may be related to the substrate unfolding process required for hydroxylation of the folded 3CA (22).

##### Pre-steady-state Studies of the Reactions of FIH·Fe(II)·2OG·ARD complexes with O_2_

**FIGURE 9.**
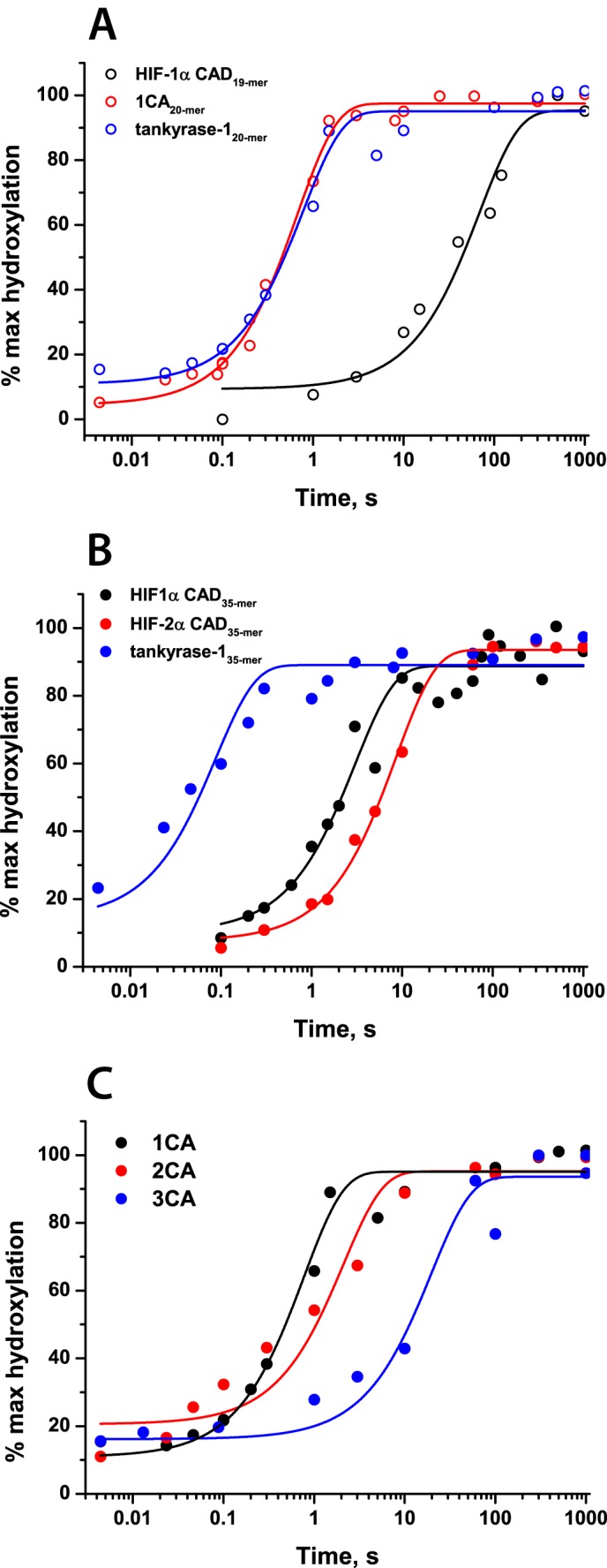
**Rapid quench-flow experiments showing FIH-catalyzed hydroxylation of HIF-1/2α CAD and ARD substrates.** Reaction mixtures containing anaerobic 0.5 mm apo-FIH, 0.4 mm Fe(II), 5 mm 2OG, 1 mm peptide in HEPES 50 mm (pH 7.5) were rapidly mixed with O_2_-saturated buffer at 5 °C in a 1:1 ratio and then quenched with 1% CF_3_CO_2_H at defined time points. The data were fitted with the function, *y* = *a*·(*1* − *exp*(−*bx*)) + *c*. Hydroxylation levels were assessed by MALDI-TOF-MS. *A*, hydroxylation of HIF-1α CAD, 1CA, and tankyrase-1 peptide substrates (19- and 20-mers); *B*, hydroxylation of HIF-1α, HIF-2α, and tankyrase-1 35-mer peptide substrates; *C*, hydroxylation of 1CA, 2CA, and 3CA. Data plotted were cumulatively acquired over the course of 1–3 separate experiments.

**FIGURE 10.**
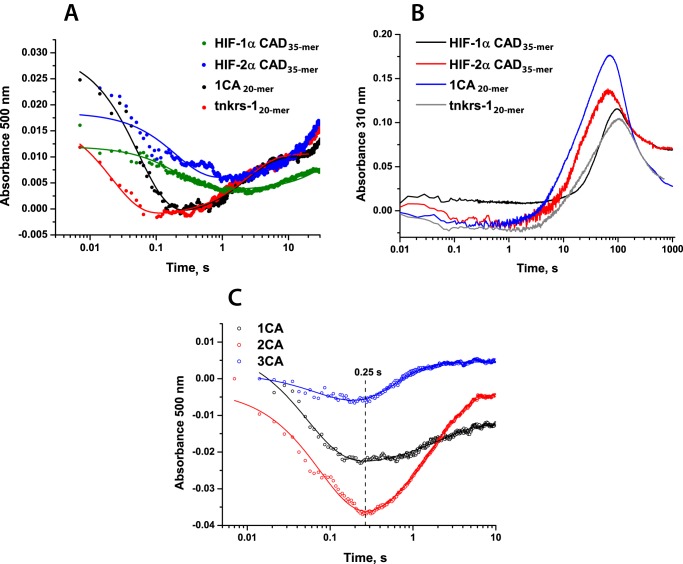
**Stopped-flow UV-visible absorbance changes upon FIH-catalyzed hydroxylation of HIF-1/2α CAD and ARD substrates.** Reaction mixtures containing anaerobic 0.5 mm apo-FIH, 0.4 mm Fe(II), 5 mm 2OG, and 1 mm peptide in HEPES 50 mm (pH 7.5) were rapidly mixed with O_2_-saturated buffer at 5 °C in 1:1 ratio and analyzed using a photodiode array detector. The data were fitted with a double exponential function. *A*, decay at 500 nm (representing enzyme·substrate complex depletion) observed upon FIH-catalyzed hydroxylation of ARD substrates compared with HIF-1/2α CAD. *B*, formation and degradation of 310 nm species observed upon FIH-catalyzed HIF-1/2α CAD and ARD hydroxylation. *C*, decay at 500 nm observed upon FIH-catalyzed hydroxylation of 1CA, 2CA, and 3CA substrates.

To further investigate the difference in the O_2_ dependence of the FIH-catalyzed hydroxylation of HIF-α and ARD substrates, stopped-flow and rapid quench-flow experiments were performed. Interestingly, the determined pre-steady-state kinetic parameters suggested markedly faster O_2_-initiated reaction rates in the presence of ankyrin rather than HIF-α CAD substrates. Upon reaction of FIH·Fe(II)·2OG·1CA_20-mer_ and FIH·Fe(II)·2OG·tnkrs-1_20-mer_ complexes with O_2_, rate constants of product accumulation were determined to be 1.3 ± 0.2 and 1.5 ± 0.1 s^−1^, respectively (*i.e.* ∼10-fold faster than the reaction with HIF 35-mer substrates) (Fig. 9). Rates of succinate production were also determined and showed similar relative changes (determined rate constants are summarized in Table 5 and supplemental Fig. S6); for both HIF-α isoforms as well as ankyrin substrates, the reaction was fully coupled. In previously reported steady-state kinetic studies (43), when the length of the peptide substrate was increased from 20-mer to 34-mer for ankyrin peptides, a ∼100-fold increase in substrate affinity was observed. O_2_ initiation of the FIH reaction with a 35-mer tankyrase-1 peptide resulted in product formation at a rate ∼7-fold faster than for the 20-mer (determined rate constants 1.5 ± 0.1 s^−1^ for tnkrs-1_20-mer_ hydroxylation, 11 ± 3 s^−1^ for tnkrs-1_35-mer_ hydroxylation). Consistent with faster hydroxylation, stopped-flow UV-visible absorbance experiments revealed that an absorbance minimum at 500 nm was reached faster for ankyrin substrates compared with HIF-α substrates (Fig. 10*A*). As for FIH-catalyzed HIF-α CAD hydroxylation, a transient species absorbing at 310 nm was observed subsequent to FIH-catalyzed ARD hydroxylation (Fig. 10*B*) (*i.e.* accumulation of an Fe(IV)-oxo intermediate was not observed (at 320 nm) in FIH catalysis).

**TABLE 5:** **Pre-steady-state kinetic parameters of FIH-catalyzed ARD hydroxylation** Conditions were as follows. 0.5 mm apo-FIH, 0.4 mm Fe(II), 5 mm 2OG, and 1 mm substrate in 50 mm HEPES (pH 7.5) were rapidly mixed with O_2_-saturated buffer at 5 °C in a 1:1 ratio. ND, not determined.

Substrate	Stopped-flow, 500 nm		Rapid quench-flow	
	*k*_fall phase_	*k*_rise phase_	*k*_product_	*k*_succinate_
	*s*^−*1*^	*s*^−*1*^	*s*^−*1*^	*s*^−*1*^
1CA	17.6 ± 0.1	0.4 ± 0.1	1.3 ± 0.2	0.8 ± 0.3
2CA	11.9 ± 0.2	0.6 ± 0.1	0.5 ± 0.1	ND
3CA	10.5 ± 0.1	1.7 ± 0.3	0.051 ± 0.001	ND
tnkrs-1_20-mer_	16.7 ± 0.2	0.4 ± 0.2	1.5 ± 0.1	1.0 ± 0.3
tnkrs-1_35-mer_	14.8 ± 0.2	0.27 ± 0.03	11 ± 3	10 ± 3

To investigate the effects of folding of ankyrin repeats on the pre-steady-state kinetics of the reaction with O_2_, the pre-steady-state parameters of the reaction with 1CA were compared with those with 2CA and 3CA (Table 5). In contrast to the effect of lengthening of the tnkrs-1 substrate, increasing the length of the consensus ankyrin repeat substrate reduced the rate constant of product accumulation, particularly for the 3CA folded substrate (0.051 ± 0.001 s^−1^ for 3CA compared with 1.3 ± 0.2 s^−1^ for 1CA), implying that ankyrin repeat folding/unfolding may have an effect on the rate of the reaction with O_2_ (Fig. 9 and Table 5). It is interesting to note that the 3CA substrate has both a significantly decreased rate of product accumulation and a significantly increased *K*_*m*_^app^(O_2_) compared with the shorter ankyrin substrates.

UV-visible stopped-flow analyses showed that the rate of the absorbance decay at 500 nm decreased with increased number of consensus ankyrin repeats (Fig. 10*C*, 17.6 ± 0.1, 11.9 ± 0.2, and 10.5 ± 0.1 s^−1^ for 1CA, 2CA, and 3CA, respectively). It is possible that the differences in FIH kinetics for the longer 3CA substrate may be due, at least in part, to the requirement for 3CA to unfold for effective catalysis (21, 44). This unfolding may be necessary for productive access of O_2_ to the active site.

It is notable that in the presence of the 3CA substrate, the rise in the absorbance at 500 nm was relatively fast (1.7 s^−1^), whereas the rate of product accumulation was slow (0.051 s^−1^). Whether or not this absorbance at 500 nm reflects reformation of an enzyme·substrate complex (32) or off-pathway accumulation of Fe(III) (42), this result raises the possibility that the reaction of FIH·Fe(II)·2OG·3CA with O_2_ is rapid but that the hydroxylated product needs to (partially) fold before it is released. This proposal is consistent with the observation that the 2CA and 1CA substrates do not fold to form the consensus ankyrin repeat structure in solution (22).

## Discussion

Hypoxia and the resultant response mediated by HIF are characteristic of multiple diseases, including some cancers (56, 57). The available evidence to date indicates that the number of direct and physiologically relevant interfaces between O_2_ and the HIF system that act in a chronic hypoxia-sensing capacity may be rather limited, with clear evidence currently only for the HIF hydroxylases themselves acting as hypoxia sensors, at least with respect to the HIF system itself. Large scale clinical trials are ongoing with PHD inhibitors for the treatment of anemia and hypoxia-related disorders (57, 58). Thus, understanding the context-dependent roles of the HIF hydroxylases is of current clinical relevance. Our results clearly reveal striking differences between the reactivity of two of the HIF hydroxylases with respect to O_2_: PHD2, the most important of the human PHDs, and FIH. The data are consistent with prior less detailed kinetic analyses (10, 43) and, importantly, with cellular observations (39) that FIH is more active at lower O_2_ concentrations than the PHDs. The complexities of the HIF hypoxic sensing system, as are likely present in any pleiotropic eukaryotic sensing mechanism acting at transcriptional and other levels (*e.g.* variations in protein levels and location), mean that we are very far from quantitative correlations between kinetic studies with isolated components and the “in cell” sensors. Nonetheless, the qualitative correlation between the relative sensitivities of PHD2 and FIH catalysis to changes in O_2_ availability with isolated components and in cells is striking.

Previous studies of the pre-steady-state kinetics of PHD2 showed very slow O_2_-mediated initiation of CODD HIF-1α hydroxylation (38, 42). In addition to the slower rate of PHD-dependent HIF hydroxylation in response to changed O_2_ concentrations, this observation was proposed to be related to the biological role of PHD2 as an O_2_ sensor in cells (39). Steady-state kinetic studies of O_2_ dependence have shown that *K*_*m*_^app^(O_2_) values for FIH are lower compared with PHD2 (10, 43); our results support these findings. Of note, we also observed a significantly faster O_2_-initiated reaction of FIH than of PHD2 under pre-steady-state conditions, consistent with our proposal that the slow reaction of PHD2 with O_2_ is linked to its ability to act as an O_2_ sensor, as reflected by its *K*_*m*_^app^(O_2_) value (10, 11, 40–42).

The results also reveal that FIH manifests mechanistic similarity to PHD2 in its lack of accumulation of the reactive Fe(IV)-oxo intermediate, which possesses distinctive spectroscopic features absorbing at 320 nm (32). This observation suggests that in terms of the rate-limiting steps of catalysis, FIH is similar to PHD2, rather than other 2OG oxygenases, such as TauD and vCPH (32, 36). Further, solvent isotope effect studies of FIH-catalyzed HIF hydroxylation have indicated that the rate-limiting step occurs after dissociation of the iron-bound water molecule from the active site and probably includes the O_2_ activation step (59), similar to that observed for PHD2 (60). This probably contributes to a degree of restraint with respect to O_2_ activation subsequent to substrate binding (binding of an Fe(II)-coordinated water molecule is proposed to be weakened upon prime substrate binding to enable O_2_ binding; Fig. 1).

We also investigated the HIF substrate dependence of O_2_ kinetics for both PHD2 and FIH. PHD2 has been shown to express a preference for hydroxylation of the CODD site, compared with NODD (46, 61). However, we found in both pre-steady-state and steady-state experiments that O_2_-initiated reaction rates are independent of the nature of the prime substrate. Faster O_2_-initiated decay of the 520 nm-absorbing enzyme·Fe(II)·2OG·substrate complex was observed in our stopped-flow experiments for HIF-2α CODD hydroxylation compared with HIF-1α CODD, suggesting faster O_2_ activation for the former. However, we did not observe any difference in the pre-steady-state time course of product accumulation for HIF-1/2α CODD, and the determined *k*_cat_^app^ values for both substrates were the same within error (0.060 ± 0.006 and 0.069 ± 0.006 s^−1^ for HIF-1α and HIF-2α CODD, respectively). Thus, although the initial step of O_2_-initiated decay of the 520 nm-absorbing species is faster for the reaction with HIF-2α CODD, the overall effect is not rate-limiting for the reaction, and steady-state parameters with respect to O_2_ are not affected.

FIH is seemingly a remarkably promiscuous oxygenase, being able to catalyze hydroxylation of Asn residues in both HIF and ARDs (19). Whereas the O_2_ dependence of HIF-1α *versus* HIF-2α was not significantly different for FIH catalysis, our results provide evidence that *K*_*m*_^app^(O_2_) values of FIH are lower with respect to ankyrin substrates, compared with HIF substrates, consistent with reported studies on Notch1–3 (43). Notably, the pre-steady-state rate of reaction of FIH with O_2_ was much faster in the presence of (most) ankyrin substrates than HIF substrates (*e.g.* 0.33 and 11 s^−1^ for HIF-1α CAD_35-mer_ and tnkrs-1_35mer_, respectively), again supporting our proposal that the rate of the O_2_-initiated reaction under pre-steady-state conditions correlates with the steady-state *K*_*m*_^app^(O_2_) parameter.

Whereas the 1CA and 2CA FIH substrates are not folded in solution, 3CA possesses a defined secondary structure (22) (note that these are consensus (*i.e.* averaged) ARD sequences rather than specific natural ARD substrates). The results imply that the secondary structure of FIH ankyrin repeat substrates can affect the rate of reaction with O_2_. Previous studies have suggested that whereas for HIF-α CAD, the secondary structure does not affect the efficiency of the FIH-catalyzed hydroxylation, for ankyrin repeat substrates, unfolding of the substrate is required prior to hydroxylation (44, 62). It has also been proposed that the difference in the efficiency of FIH-catalyzed ARD hydroxylation is related to the stability of the ARD protein fold (21). A protein with four contiguous consensus ankyrin repeats was found not to be hydroxylated by FIH, whereas 3CA is a substrate (21). The natural ARD proteins, Notch and IκBα, contain more than six ARDs and are known to be relatively efficient substrates for FIH (43, 62), including in cells (18). Thus, the number of contiguous ARDs in a protein does not necessarily correlate with the hydroxylation efficiency, which is also related to local stability and sequence of the hydroxylated region (21, 22). (Note also that FIH probably interacts with ARDs that it does not hydroxylate (22).) The observation that ARD folding affects FIH catalysis is in agreement with our observations, where a slower reaction with a folded ankyrin substrate was observed. It suggests that the unfolding step, which occurs prior to hydroxylation, affects the kinetics of ARD hydroxylation. If O_2_ reactivity is limited by access to the active site, this may rationalize the observed slower rate for the 3CA substrate. This may also rationalize the overall observation of faster kinetics with respect to O_2_ for FIH than for PHD2; PHD2 structural studies revealed a “tight” active site relative to that of FIH (45, 63). Further, if the nature of the enzyme-substrate interaction affects O_2_ access to the active site, this raises the possibility that alternative substrates for the HIF hydroxylases may have different O_2_ sensitivity profiles.

The influence of enzyme-substrate interactions on the reaction of FIH with O_2_ is of interest in terms of understanding the prime biological function of FIH. Although FIH is involved in hypoxia sensing in cells (25), the precise importance of the role of ARD hydroxylation in the hypoxic response is not well understood. Taking into account the abundance of ARD substrates in cells, our results indicate that FIH has the potential to hydroxylate ARD substrates under conditions of low O_2_ availability, where its ability to hydroxylate HIF substrates may be substantially diminished. Thus, at least on a short time scale (*i.e.* tens of minutes) FIH-catalyzed ARD hydroxylation may not directly contribute in a sensing capacity to the hypoxic response. However, it has the possibility of contributing to the context-dependent regulation of the hypoxic response on a longer time scale (including by enabling memory effects (64)) by altering the kinetics of FIH HIF-α catalysis in cells by regulating the fraction of FIH free to hydroxylate HIF-α, because (at least some) hydroxylated ARDs bind less tightly to FIH than unhydroxylated ARDs (19, 44).

## Author Contributions

H. T. and E. F. designed the study and wrote the paper. H. T. expressed and purified proteins and performed all of the kinetic studies. A. P. H. assisted with purification of 2CA and 3CA and with protein LC/MS. E. A. H. performed CD studies on 2CA and 3CA. N. D. L. and J. S. O. M. assisted with succinate determination by LC/MS. H. B. K. performed MALDI-TOF/TOF-MS/MS experiments. C. J. S. critically revised the manuscript. All authors analyzed the results and approved the final manuscript.

## Supplementary Material

Supplemental Data

## Footnotes

3 The abbreviations used are:

HIF: hypoxia-inducible factor
2OG: 2-oxoglutarate
ARD: ankyrin repeat domain
CA: consensus ankyrin
CAD: C-terminal transactivation domain
CODD: C-terminal oxygen-dependent degradation domain
FIH: factor inhibiting HIF
NODD: N-terminal oxygen-dependent degradation domain
PHD: prolyl hydroxylase domain
CREB: cAMP-responsive element-binding protein
tnkrs: tankyrase.
