# Supplementary material for: Kinetic Investigations of the Role of Factor Inhibiting Hypoxia-inducible Factor (FIH) as an Oxygen Sensor
Source: J Biol Chem. 2015 Jun 25;290(32):19726–42. doi: 10.1074/jbc.M115.653014 (PMC4528135; doi:10.1074/jbc.M115.653014)
Supplement: Supplemental Data [file supp_290_32_19726__index.html]

Kinetic Investigations of the Role of Factor Inhibiting Hypoxia-Inducible Factor (FIH) as an Oxygen Sensor — Kinetic Investigations of the Role of Factor Inhibiting Hypoxia-inducible Factor (FIH) as an Oxygen Sensor — Kinetic Studies on the Role of FIH as an Oxygen Sensor — Supplemental Data 

# Kinetic Investigations of the Role of Factor Inhibiting Hypoxia-inducible Factor (FIH) as an Oxygen Sensor

## Supplemental Data

- Tarhonskaya et al Supplementary Information (.docx, 1.5 MB) - Tarhonskaya et al JBC/2015/653014 v3 Supplementary Information
