# Supplementary material for: Kinetic Investigations of the Role of Factor Inhibiting Hypoxia-inducible Factor (FIH) as an Oxygen Sensor
Source: J Biol Chem. 2015 Jun 25;290(32):19726–42. doi: 10.1074/jbc.M115.653014 (PMC4528135; doi:10.1074/jbc.M115.653014)
Supplement: Supplemental Data [file supp_M115.653014_jbc.M115.653014-1.docx]

**Supplementary Information**

**Kinetic Investigation on the Role of Factor Inhibiting Hypoxia-Inducible Factor (FIH) as an Oxygen Sensor**

***Kinetic Modelling***

Hanna Tarhonskaya^1^, Adam P. Hardy^1^, Emily A. Howe^1^, Nikita D. Loik^1†^, Holger B. Kramer^2^, James S. O. McCullagh^1^, Christopher J. Schofield^1^, Emily Flashman^1^*

*^1^Chemistry Research Laboratory, University of Oxford, 12 Mansfield Road, Oxford, OX1 3TA, United Kingdom.*

*^2^OXION Proteomics Facility, Department of Physiology, Anatomy and Genetics, University of Oxford, South Parks Road, Oxford, OX1 3QX, United Kingdom.*

**Table of contents**

[Figure S1. Peptide substrate *K_m_^app^* determination for PHD2-catalyzed hydroxylation. S2](#_Toc414573728)

[Figure S2. 2OG *K_m_^app^* determination for PHD2-catalyzed hydroxylation. S3](#_Toc414573729)

[Figure S3. Determination of kinetic parameters for FIH-catalyzed HIF-1/2α CAD hydroxylation. S4](#_Toc414573730)

[Figure S4. O_2_-dependence of the FIH-catalyzed hydroxylation of HIF-1α CAD peptides (19-mer vs. 35-mer). S5](#_Toc414573731)

[Figure S5. Rapid quench-flow experiments showing PHD2-catalyzed HIF-1/2α hydroxylation. S6](#_Toc414573732)

[Figure S6. Rapid quench-flow experiments showing FIH-catalyzed hydroxylation of HIF-1/2α and ARD peptides. S7](#_Toc414573733)

[Figure S7. Determination of kinetic parameters for FIH-catalyzed ankyrin domain repeat (ARD) hydroxylation. S8](#_Toc414573734)

[Figure S8. Kinetic parameters of the FIH-catalyzed hydroxylation of (A, B) 2CA and (C, D) 3CA. S9](#_Toc414573735)

**Supplementary Figures**

**
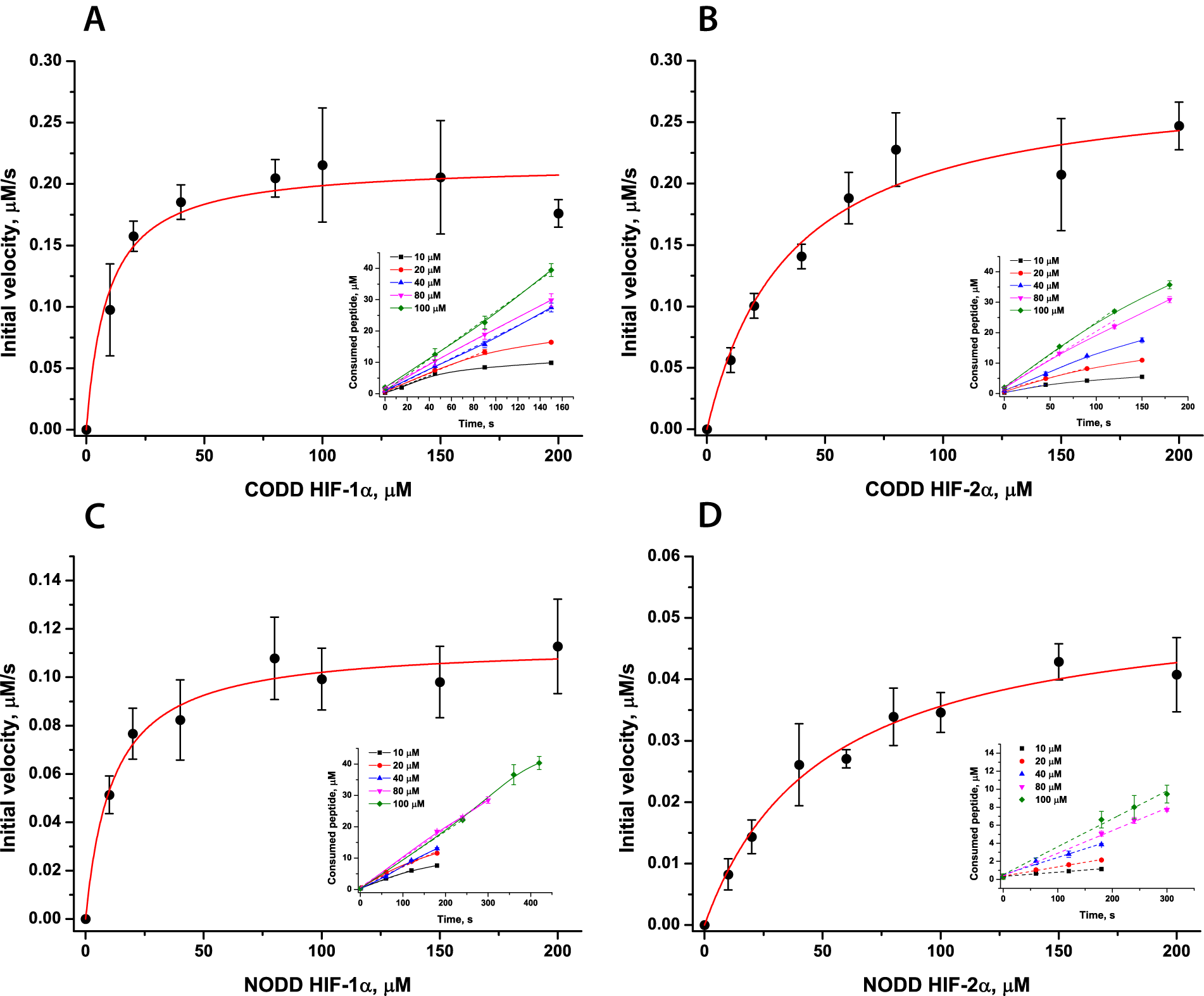
**

Figure S1. Peptide substrate *K_m_^app^* determination for PHD2-catalyzed HIF-1/2α hydroxylation. The following peptide substrates were used: **A.** CODD HIF-1α, **B.** CODD HIF-2α, **C.** NODD HIF-1α, **D.** NODD HIF-2α. Conditions: samples containing 4 µM PHD2, peptide (varied), 300 µM 2OG, 50 µM Fe(II), 4 mM L-ascorbate in 50 mM Tris·HCl (pH 7.5) buffer were incubated at 37 °C. Peptide hydroxylation was analyzed by MALDI-TOF-MS. Error bars represent SD of triplicate assays. Insets show representative time courses at different concentrations of peptide. Peptide sequences are defined in Table 1 in the main text.


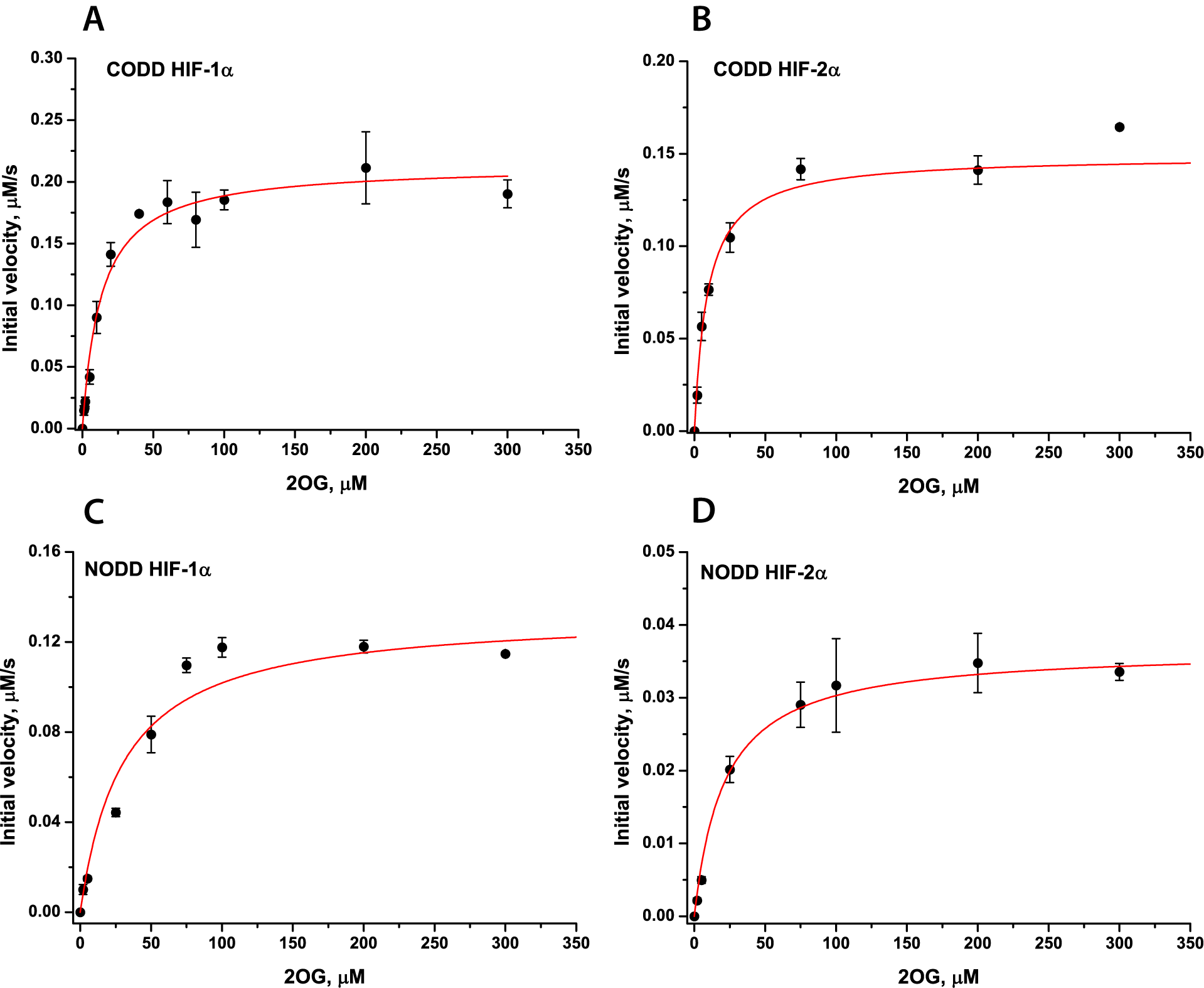


Figure S2. 2OG *K_m_^app^* determination for PHD2-catalyzed HIF-1/2α hydroxylation. The following peptide substrates were used: **A.** CODD HIF-1α, **B.** CODD HIF-2α, **C.** NODD HIF-1α, **D.** NODD HIF-2α Conditions: samples containing 4 µM PHD2, 100 µM peptide, 2OG (varied) 50 µM Fe(II), 4 mM L-ascorbate in 50 mM Tris·HCl (pH 7.5) buffer were incubated at 37 °C. Peptide hydroxylation was analyzed by MALDI-TOF-MS. Error bars represent SD of triplicate assays..


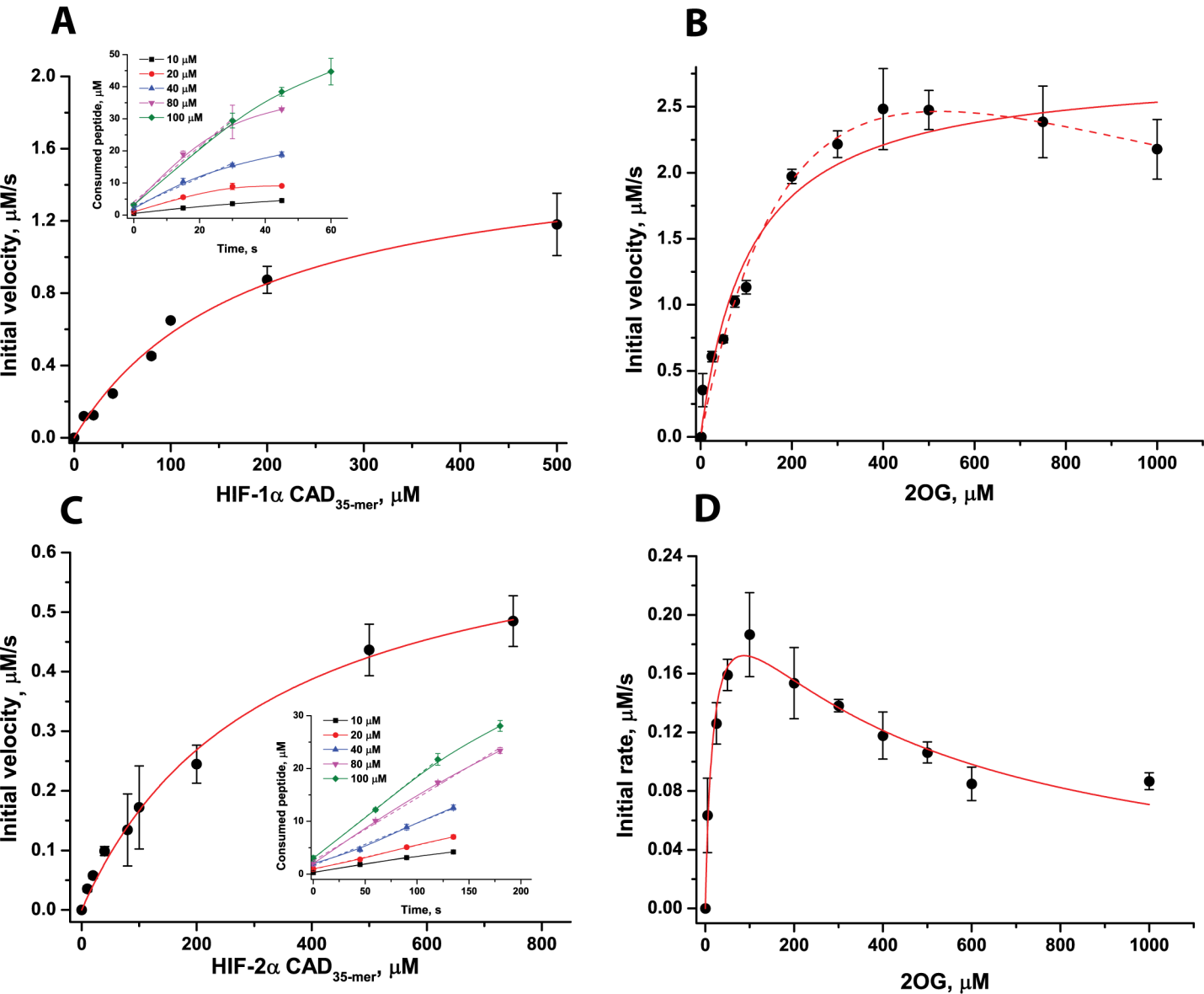


Figure S3. Determination of kinetic parameters for FIH-catalyzed HIF-1/2α CAD hydroxylation. **A.** HIF-1α CAD_35-mer_  *K_m_^app^* determination; **B.** 2OG *K_m_^app^* determination in the presence of HIF-1α CAD_35-mer_; Fitting with a substrate inhibition model (dashed line) gives *K_m_^app^*(2OG)=300±100 μM, *K_i_*=900±400 μM. **C.** HIF-2α CAD_35-mer_ *K_m_^app^* determination; substrate inhibition is apparent at concentrations of peptide >1 mM (data not shown). **D.** 2OG *K_m_^app^* determination in the presence of HIF-2α CAD_35-mer_ (data fitted with substrate inhibition model). Conditions: 5 µM FIH, 50 µM Fe(II), 1 mM L-ascorbate, various concentrations of the peptide and 2OG. Initial rates were determined varying concentration of one of 2OG/peptide. The concentration of HIF-1/2α CAD was 500-1000 µM (if fixed) and that of 2OG was 1 mM (if fixed). Peptide hydroxylation was analyzed by MALDI-TOF-MS. Insets show representative time courses at different concentrations of peptide. Error bars represent SD of triplicate assays. Peptide sequences are defined in Table 1 in the main text.

**
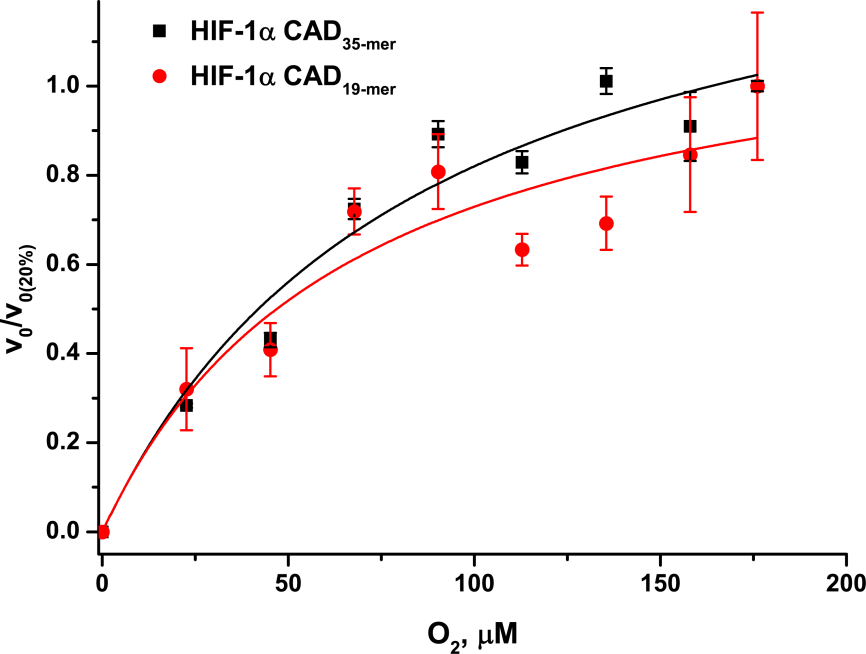
**

Figure S4. O_2_-dependence of the FIH-catalyzed hydroxylation of HIF-1α CAD peptides (19-mer vs. 35-mer). Conditions: 5 µM FIH, 500 µM peptide (peptide sequences defined in Table 1, main text), 50 µM Fe(II), 1 mM 2OG, 1 mM L-ascorbate in HEPES 50 mM (pH 7.5) were incubated at 37 °C in a hypoxic workstation under different pO_2_. The different hydroxylation levels were analyzed by MALDI-TOF-MS, and the relative initial rates (to the initial rate of the reaction at 20% O_2_) are presented. Error bars represent SD of triplicate assays.


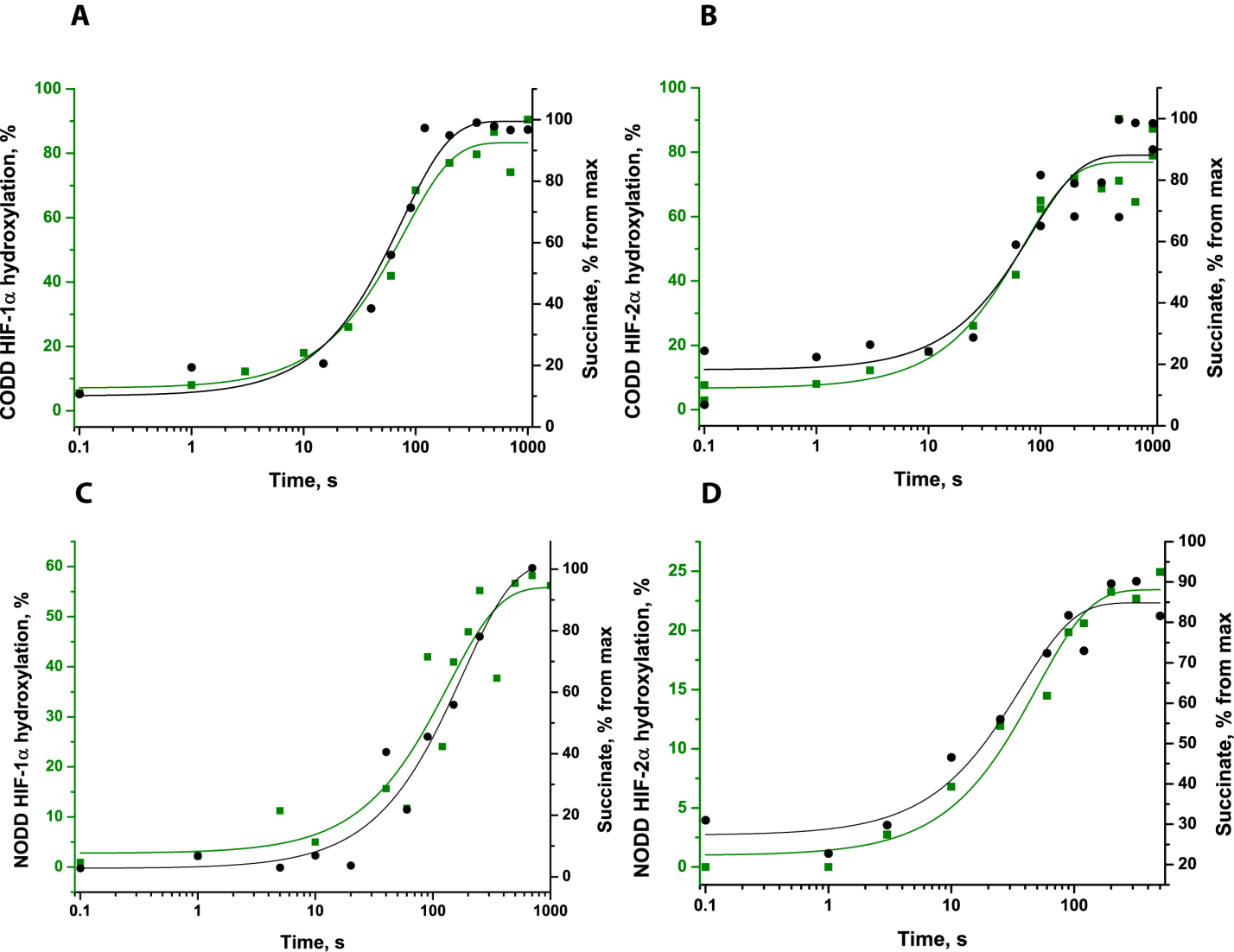


Figure S5. Rapid quench-flow experiments showing PHD2-catalyzed HIF-1/2α hydroxylation. The following peptide substrates were used: **A.** CODD HIF-1α, **B.** CODD HIF-2α, **C.** NODD HIF-1α, **D.** NODD HIF-2α (sequences defined in Table 1, main text). Reaction mixtures containing 0.8 mM apo-PHD2, 0.5 mM Fe(II), 5 mM 2OG, 1 mM peptide in HEPES 50 mM (pH 7.5) were rapidly mixed with O_2_-saturated buffer at 5 °C in 1:1 ratio and then quenched with 1% CF_3_COOH at defined time points. The data were fitted with *y=a·(1-exp(-bx))+c* function. Hydroxylation levels were assessed by MALDI-TOF-MS; succinate accumulation was analyzed by LC-MS.


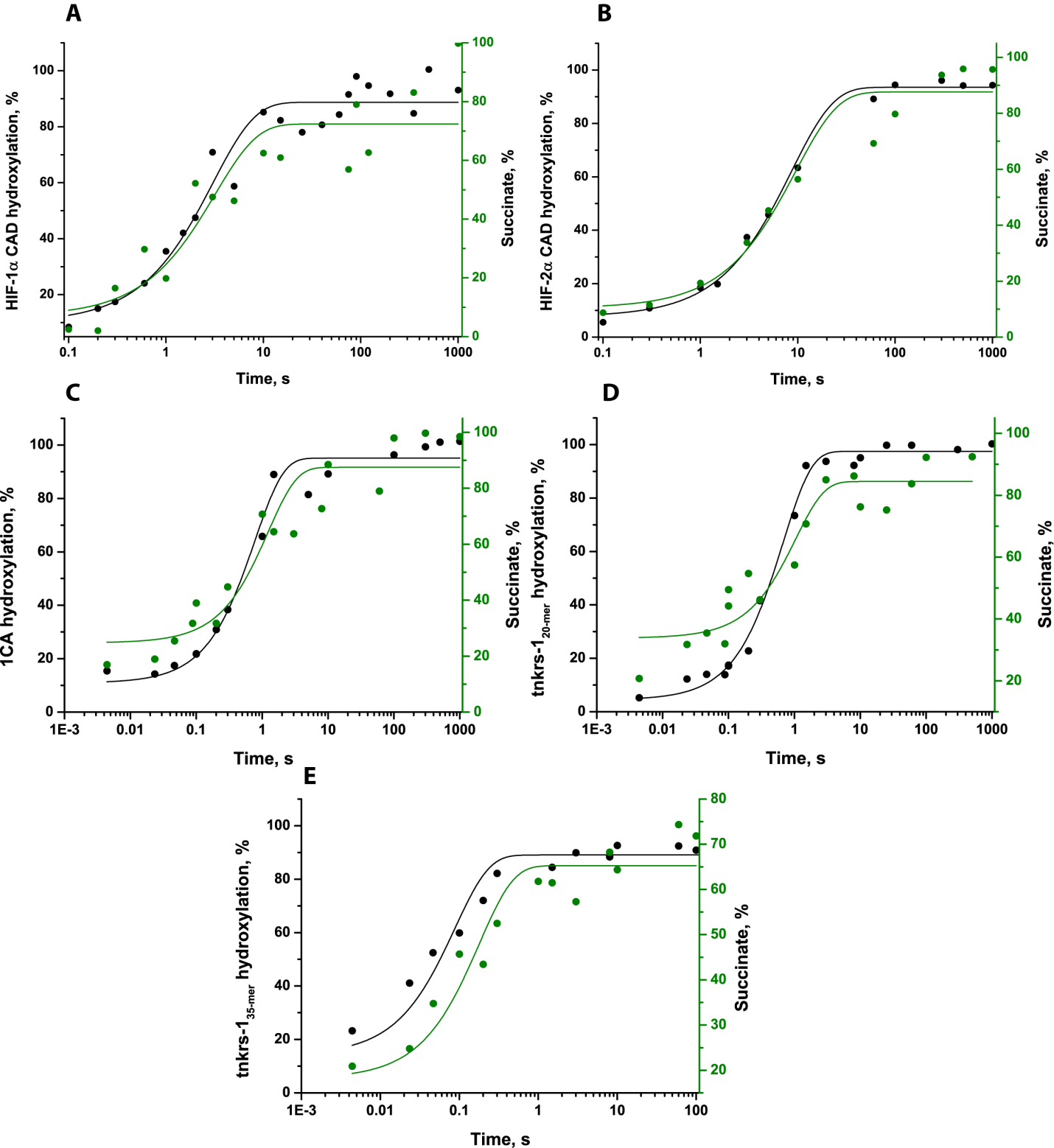


Figure S6. Rapid quench-flow experiments showing FIH-catalyzed hydroxylation of HIF-1/2α CAD and ARD peptides. The following peptide substrates were used: **A.** CAD HIF-1α 35-mer, **B.** CAD HIF-2α 35-mer, **C.** 1CA, **D.** tankyrase-1 20-mer, **E.** tankyrase-1 35-mer (sequences defined in Table 1, main text). Reaction mixtures containing 0.5 mM apo-FIH, 0.4 mM Fe(II), 5 mM 2OG, 1 mM peptide in HEPES 50 mM (pH 7.5) were rapidly mixed with O_2_-saturated buffer at 5 °C in 1:1 ratio and then quenched with 1% CF_3_COOH at defined time points. The data were fitted with *y=a·(1-exp(-bx))+c* function. Hydroxylation levels were assessed by MALDI-TOF-MS and succinate accumulation by LC-MS.


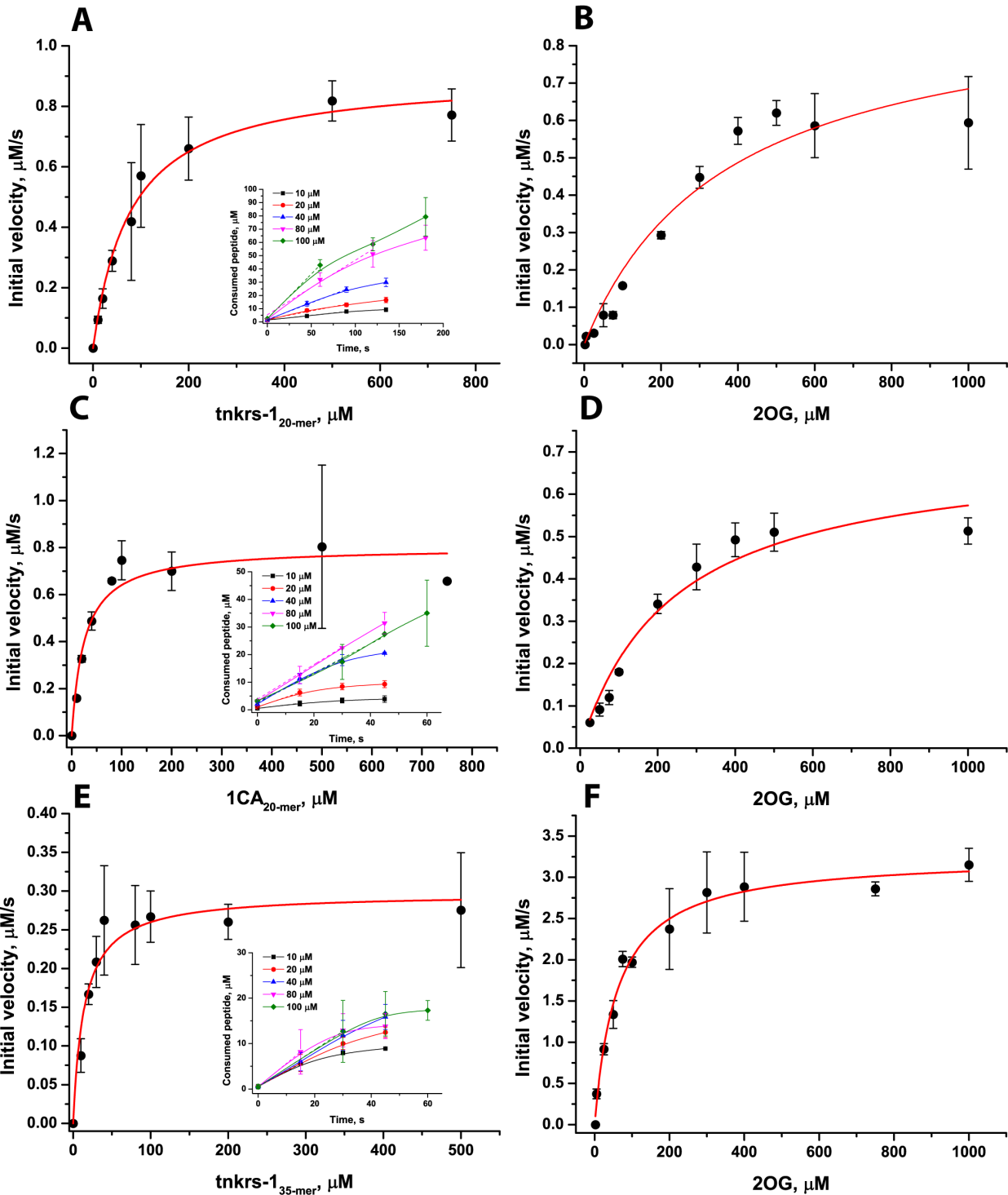


Figure S7. Determination of kinetic parameters for FIH-catalyzed ARD peptide hydroxylation. **A.** tankyrase-1 (tnkrs-1_20-mer_) *K_m_^app^* determination; **B.** 2OG *K_m_^app^* determination in the presence of tnkrs-1_20-mer_; **C.** 1 consensus anlyrin repeat (1CA_20-mer_) *K_m_^app^* determination; **D.** 2OG *K_m_^app^* determination in the presence of 1CA_20-mer_; **E.** tnkrs-1_35-mer_  *K_m_^app^* determination; **F.** 2OG *K_m_^app^* determination in the presence of tnkrs-1_35-mer_;. Conditions: 5 µM FIH, 50 µM Fe(II), 1 mM L-ascorbate, various concentrations of the peptide and 2OG. Initial rates were determined at 37 °C varying concentration of one of 2OG/peptide. Concentration of HIF-1/2α CAD was 500-1000 µM (if fixed) and 2OG was 1 mM (if fixed). Peptide hydroxylation was analyzed by MALDI-MS. Insets are showing representative time courses at different concentrations of peptide. Error bars represent SD of triplicate assays.

**
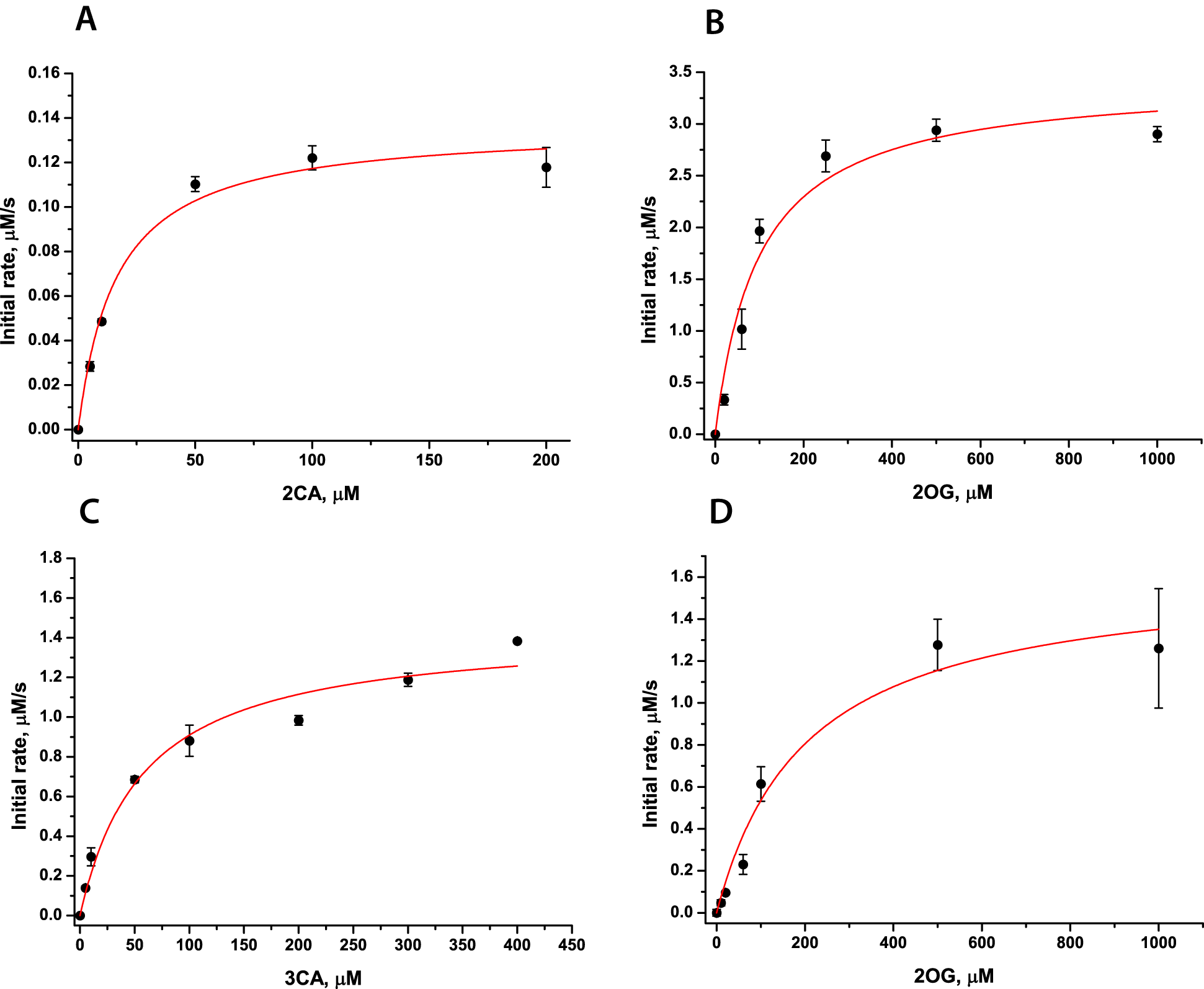
**

Figure S8. Kinetic parameters of FIH-catalyzed hydroxylation of 2CA (A,B) and 3CA (C,D) ARD substrates. Substrate sequences are defined in Table 1 in the main text. Conditions: 0.1 µM FIH (A) or 2 µM FIH (B-D), peptide/2OG (varied), 50 µM Fe(II), 1 mM L-ascorbate in 50 mM HEPES ((pH 7.5)) were incubated at 37 °C. Hydroxylation levels were analyzed by LC-MS. Error bars represent SD of triplicate assays. The differences in *V*_max_ for the same substrate reflect different FIH concentrations used in the assays.
